# Protocol of the PANEM trial: A randomized, phase III, parallel-arm, placebo-controlled, double-blind, multi-center superiority trial comparing PANcreatic EnzyMes versus placebo in adults after gastrectomy

**DOI:** 10.1371/journal.pone.0358177

**Published:** 2026-09-22

**Authors:** Kevin Thomas, Charlotte Ackmann, Anett Schmiedeknecht, Kathrin Scheibe, Tobias Kleemann, Michael Ardelt, Felix Dondorf, Olga Radulova-Mauersberger, Jasmina Kuvendjiska, Julian Hipp, Boris Jansen-Winkeln, Thomas Bley, Ulrike Wetzold, Judith Sporn, Franziska Peters, Patrick Téoule, Emrullah Birgin, David Petroff, Albrecht Hoffmeister

**Affiliations:** 1 Department of Medicine, Neurology and Dermatology, Division of Oncology, Gastroenterology, Hepatology and Pneumology, Leipzig University Medical Center, Leipzig, Germany; 2 Clinical Trial Centre Leipzig, Leipzig University, Medical faculty, Leipzig, Germany; 3 4th Medical Clinic for Gastroenterology and Rheumatology, Medical University of Lausitz - Carl Thiem, Cottbus, Germany; 4 Department of General, Visceral and Vascular Surgery, University Hospital Jena, Jena, Germany; 5 Department of Visceral, Thoracic and Vascular Surgery University Hospital Carl Gustav Carus Dresden, Dresden, Germany; 6 Department of General and Visceral Surgery, University Medical Center Freiburg, Freiburg, Germany; 7 Department of General, Visceral, Thoracic and Vascular Surgery, St. George’s Hospital Leipzig, Leipzig, Germany; 8 Department of Surgery, Medical University of Lausitz - Carl Thiem, Cottbus, Germany; 9 Department of General and Visceral Surgery, Helios Hospital Schwerin, Schwerin, Germany; 10 Department of General and Visceral Surgery, University Hospital Ulm, Ulm, Germany; University of Nebraska Medical Center College of Medicine, UNITED STATES OF AMERICA

## Abstract

**Background:**

Patients after partial or total gastrectomy suffer from malnourishment and diminished quality of life (QOL). Due to altered physiological processes after gastric surgery, coordination between chyme and digestive enzymes produced by the pancreas is affected in many ways, finally leading to maldigestion and malabsorption. These in turn promote various symptoms such as discomfort or steatorrhea. Supplementation of digestive enzymes during food intake may improve digestion and decrease nutrition-related abdominal symptoms, finally leading to higher QOL. To date, trials examining the role of digestive enzyme supplementation in patients with gastrectomy are lacking.

**Methods:**

We designed a randomized, phase III, parallel-arm, placebo-controlled, double-blind, multi-center superiority trial comparing QOL after 6 months of oral digestive enzyme supplementation vs. placebo in a sample of 188 patients after partial or total gastrectomy. Digestive enzymes or placebo are taken orally with the meals. Cancer specific QOL (physical sub-score from EORTC-QLQ-C30) at six months is the primary outcome. Secondary outcomes include other measures of QOL, weight loss, use of supplemental nutrition, and laboratory measurements related to malnutrition and metabolism.

**Discussion:**

This trial will help assess using digestive enzymes in patients with partial or total gastrectomy.

**Trial Registration:**

EU Trial Number: 2022-501944-14-00. Clinical Trial Number: NCT06058442 (Registration date: 09/08/2023).

## Background

Gastric cancer is the fifth most prevalent cancer worldwide with an incidence of almost one million new cases each year [1]. Significant improvements in outcomes have been demonstrated over time: in an analysis spanning 30 years, perioperative mortality after curative gastrectomy decreased from 8% to 1%, while median overall survival increased from 28 to 53 months [2]. Depending on the stage of the tumor, surgical resection is the indicated treatment in the majority of cases, often with curative intent, and usually combined with perioperative chemotherapy [3]. Other indications for partial or total gastrectomy comprise prophylactic gastrectomy in hereditary forms of gastric cancer, bariatric surgery or gastric ulcers that are refractory to pharmaceutical or endoscopic treatment. Despite advances in gastrointestinal surgery, malnourishment is common after gastrectomy and is associated with post-operative morbidity [4].

Anatomical changes following gastrectomy lead to altered physiological processes, i.e., the impaired coordination of release of enzymes and the altered transit of nutrients [5]. In particular, as a result of upper gastrointestinal resection, postoperative plasma concentrations of pancreatic hormones such as cholecystokinin or secretin decrease [6,7]. Among others, these processes are postulated to promote impaired coordination between gastric emptying and discharge of bilio-pancreatic enzymes, a mechanism called postcibal asynchrony. Digestion and resorption of macronutrients diminish, finally resulting in exocrine pancreas insufficiency (EPI) [8,9]. This in turn promotes discomfort caused by symptoms such as steatorrhea, weight loss, bloating and abdominal pain. According to a recent meta-analysis, prevalence of EPI after total gastrectomy is around 66% [10]. Evidence suggests that post-gastrectomy symptoms due to EPI might be underestimated in the clinical context and therefore highlights the role of pancreas or other digestive enzyme supplementation as a therapeutic target to address malnourishment, well-being and discomfort in these patients [11,12]. However, previous studies showed inconsistent results concerning pancreas enzyme replacement therapy in this population.

A small, older, double-blind crossover trial (n = 15) compared the supplementation of pancreatic enzymes with placebo in patients after gastrectomy over only 7 days to assess abdominal symptoms and stool properties and found improvements in steatorrhea and stool consistency while abdominal symptoms remained unchanged [11]. The same group performed a second double-blind randomized controlled trial about ten years later (n = 52) with 14 days of treatment and similar endpoints, but did not find meaningful or significant effects, though improvement in QOL was detected [13]. An open-label randomized trial comparing “normal diet” to supplementation of pancreatic enzymes at meals with a treatment and observation time of 12 months found no effect on body mass index (BMI), but some indication for improvements in QOL and nutritional status [14]. The main limitations of this last trial are the open label design and its low sample size (n = 43), which was based on the extremely optimistic expectation that BMI in the intervention group would be 20% higher than in the control group.

Reviews conclude that effects of pancreatic enzymes in gastrectomized patients appear to be small, but that evidence is lacking, and that trials performed were of small sample size and low quality [15]. Other authors are thus cautious in their recommendations for the routine use of pancreatic enzymes in this indication [16]. Additionally, dosing of pancreatic enzymes in patients after gastric surgery is not standardized thus leading to pragmatic dosing approaches with limited comparability [17].

Due to limited and methodologically constrained evidence on digestive enzyme therapy after gastrectomy, the **Pan**creatic  **E**nzy**m**es after Gastrectomy Trial (PANEM) was designed. The primary objective of this double-blind randomized controlled trial was to assess the effect of digestive enzyme supplementation versus placebo on QOL.

## Methods

### Setting and funding

PANEM is an investigator initiated, randomized, phase III, parallel-arm, placebo-controlled, double-blind, multi-center superiority trial. Patients will be randomized either to receive digestive enzyme therapy (NORTASE^®^) or placebo for 6 months after partial or total gastrectomy. The trial is planned to be conducted in approximately ten different hospitals in Germany.

The legal sponsor of the trial is the Leipzig University and it is financed by Repha GmbH Biologische Arzneimittel (Repha). The latter did not participate in designing the trial. Repha will not be involved in analyzing data or drafting the manuscript, though it has the right to comment on the manuscript before submission. The authors are not obligated to implement suggestions by Repha.

### Ethics approval and consent to participate

The study protocol was reviewed and approved in accordance with Regulation (EU) No. 536/2014 by the Federal Institute for Drugs and Medical Devices, Bonn, Germany, with a positive opinion from the Ethics Committee of the State of Berlin. Approval was granted on 24 April 2023 under EU Clinical Trial Number 2022-501944-14-00 and procedural reference number B_00205.

All persons participating in the conduct of the trial (sponsor, authorized representative of the sponsor, investigators, etc.) commit themselves to conduct the trial according to the trial protocol, the Declaration of Helsinki of the World Medical Association (in its current version), as well as the EU CTR 536/2014 and the ICH guideline for Good Clinical Practice ICH E6(R2) (EMA/CHMP/ICH/135/1995) issued in June 2017.

The SPIRIT checklist and the full study protocol are provided as Supporting Information (S1 Checklist and S2 Protocol).

### Eligibility criteria

Having provided written informed consent, the PANEM trial enrolls all adult patients who have undergone total or partial gastrectomy, irrespective of the underlying indication for surgery. Given that gastric cancer represents the predominant indication for gastrectomy, the majority of the study population is expected to comprise patients with oncological diagnoses. However, patients who have undergone gastrectomy for other etiologies are equally eligible for inclusion. Generally, subjects are enrolled in the trial by the local investigator or authorized medical staff at the trial sites.

Table 1 summarizes the exclusion criteria as stated in the latest version of the study protocol (S2 Protocol).

**Table 1:** Exclusion criteria for the PANEM trial.

• Indication for pancreas enzyme therapy
• Gastrectomy with palliative intention
• Union for International Cancer Control (UICC) Stage IV gastric malignancy unless surgery is planned according to new guidelines in oligometastatic disease
• Malnutrition of other etiology
• Life expectancy less than 12 months
• Known lactose intolerance
• Known hereditary galactose intolerance
• Patients on alpha-glucosidase inhibitors
• Acute pancreatitis
• Acute episode of chronic pancreatitis
• Known hypersensitivity to moulds (mould allergy) or any other ingredient of NORTASE® • Patients under legal supervision or guardianship • Patients who are dependent on the investigator or the medical staff of the trial team or the coordinating investigator or the sponsor • Fertile women (within two years of their last menstruation) without appropriate contraceptive measures while participating in the trial • Pregnant or nursing women • Suspected lack of compliance • Patients who were already enrolled in the trial

Potential patients will be informed about the PANEM trial during their inpatient stay before or after gastrectomy, but no later than three days after beginning oral nutrition. Informed consent will be obtained by authorized staff at the trial sites.

Procedures performed solely for the clinical trial can be performed only after obtaining informed consent.

### Trial medication

The investigational product, i.e., NORTASE^®^, and placebo will be manufactured, packaged and labelled by Repha and contracted third-party manufacturers according to the standards of Good Manufacturing Practice. Each hard capsule of NORTASE^®^ contains fungal-derived lipase (7,000 FIP units), protease (minimum 54 FIP units), and amylase (minimum 700 FIP units), where FIP denotes the standardized enzyme activity units defined by the Fédération Internationale Pharmaceutique. NORTASE^®^ and placebo are dispensed with 100 capsules per bottle. For each patient, the entire trial medication is packed individually in a box, i.e., this box contains a total of 28 bottles of NORTASE^®^ or placebo, ideally reaching up to the end of individual medication after 6 months.

According to the “Summary of Product Characteristics” (SmPC), participants should take 5–15 capsules of NORTASE^®^ per day, distributed with meals (1–3 capsules per meal). Gastrectomized patients typically eat five or more small meals per day instead of three.

Patients start administration in the hospital on day 0 with an initial dose of 5 (meals) x 2 capsules taken with meals, i.e., 10 capsules per day. If necessary, the dosage can be increased to 5 (meals) x 3 capsules, i.e., 15 capsules per day after three weeks.

It is possible to reduce the dosage without stopping the treatment. The capsules dissolve quickly even in a pH neutral liquid and are generally ingested whole with sufficient liquid (water, juice or warm tea) during a meal or snack but can also be opened beforehand. Participants receive structured instructions on the proper administration of the study medication during their first study visit. This ensures that all participants, regardless of group allocation, receive uniform guidance on how and when to take the medication.

### Concomitant care

Standard treatment will be started/continued in both treatment groups, and should follow national guidelines, or international guidelines if national ones are not available. Standard treatment will often include chemotherapy in this patient population (cf. Statistical analysis).

### Objectives and outcomes

The primary objective of this trial is to test for improved cancer specific QOL in gastrectomized patients after six months of digestive enzyme replacement therapy.

Secondary objectives comprise enhancement of nutritional status, weight loss and laboratory measurements related to malnutrition and metabolism after digestive enzyme supplementation in these patients.

Cancer specific QOL will be evaluated using the physical sub-score of the EORTC Core Quality of Life Questionnaire (EORTC QLQ-C30), an established international instrument, which has been validated in a German population [18,19]. The EORTC QLQ-C30 questionnaire contains of 30 questions and assesses the QOL in patients with cancer across different tumor entities multidimensionally via 10 subscales on a numeric rating scale from zero (= not at all) to four (= very) [19]. Two items consist of a seven-stage numeric scale evaluation of health status and QOL within the last 14 days.

Secondary endpoints comprise blood and stool parameters as well as nutrition-related aspects and weight loss. Hence, patients will be asked about the typical number of meals they have per day and about nutritional supplementation.

Moreover, anthropometric and demographic as well as medical data including medical history, concomitant medication and vital signs are assessed at each visit. Finally, the proportion of patients who receive supplemental nutrition or who have more than 10% weight loss at six months is calculated. Blood sample analysis at the individual visits include serum hemoglobin, total protein, albumin, random glucose, HbA1c, cholesterol, vitamins A, B12, D, E and K. Optionally, at selected trial sites fecal elastase-1 test will be performed.

In addition to the EORTC QLQ-C30, two further validated instruments are employed as secondary outcome measures. The Gastrointestinal Quality of Life Index (GIQLI) is a 36-item questionnaire covering gastrointestinal symptoms, physical, emotional, and social function, and has been widely used to assess outcomes after gastrointestinal surgery, including gastrectomy for gastric cancer [20,21]. The EORTC QLQ-STO22 is a gastric cancer-specific module designed to supplement the EORTC QLQ-C30, addressing symptoms such as dysphagia, eating restrictions, pain, reflux, and anxiety, and has been validated across various settings [22,23].

Even though no intervention-related effect is assumed, Patients will be contacted at regular interval to assess mortality.

### Individual timeline, assessments, compliance and premature termination

**Fig 1:**
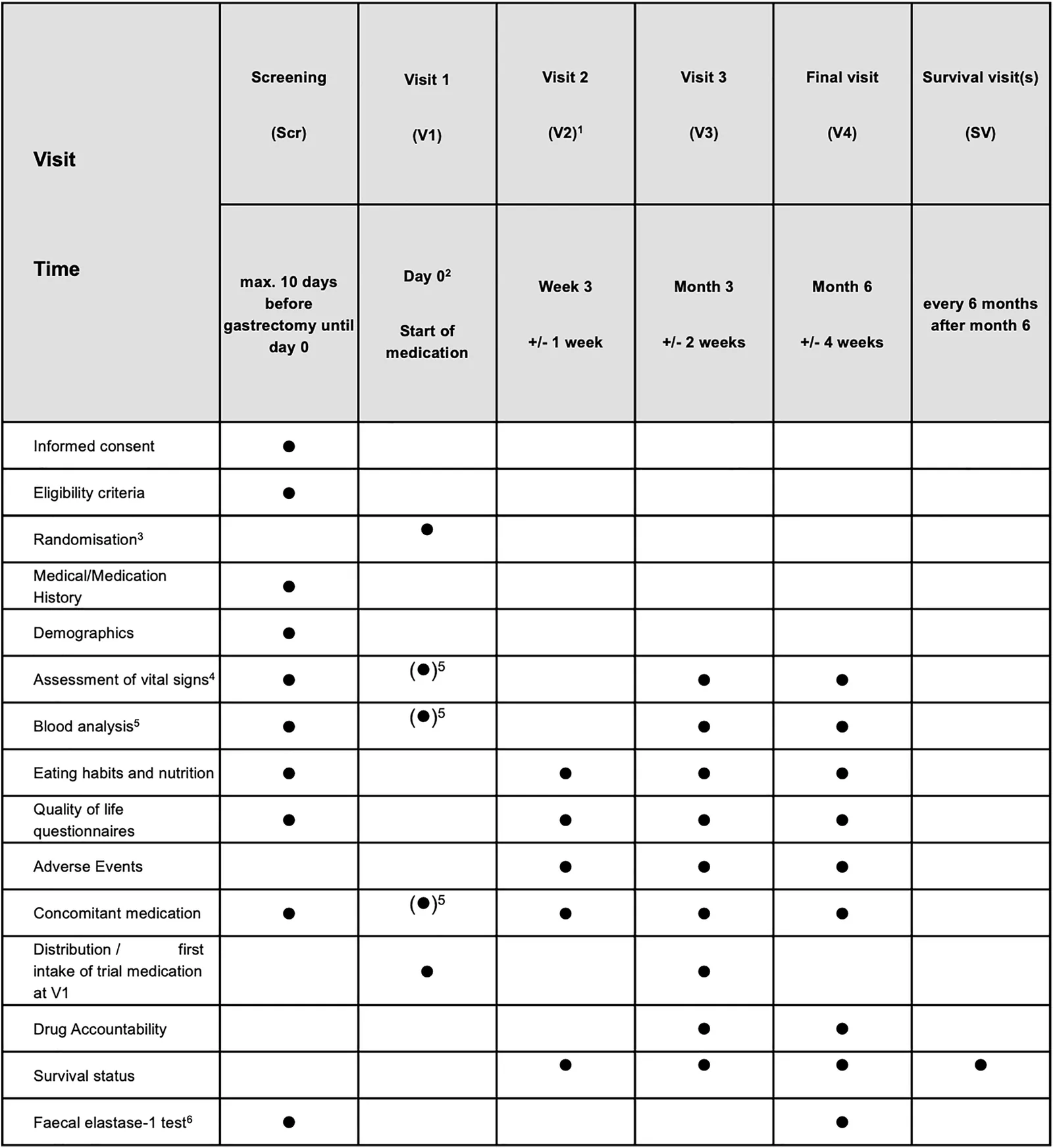
Schedule of assessments and procedures. ^1^ if necessary, only by phone. ^2^ ideally with the begin of oral nutrition, max. 3 days after begin of oral nutrition; ^3^ after gastrectomy, immediately before or at day 0; ^4^ arterial blood pressure, heart rate, temperature, body weight; ^5^ if screening visit occurs on day 0 (visit 1), these examinations are only performed once; ^6^ incl. serum hemoglobin, total protein, albumin at all visits, where glucose, HbA1c, cholesterol, vitamins A, B12, D, E and K only need to be measured once, either for visit Scr or V1, and V3 + V4, pregnancy test only at screening; ^7^ optional, all patients at selected trial sites.

**Fig 2:**
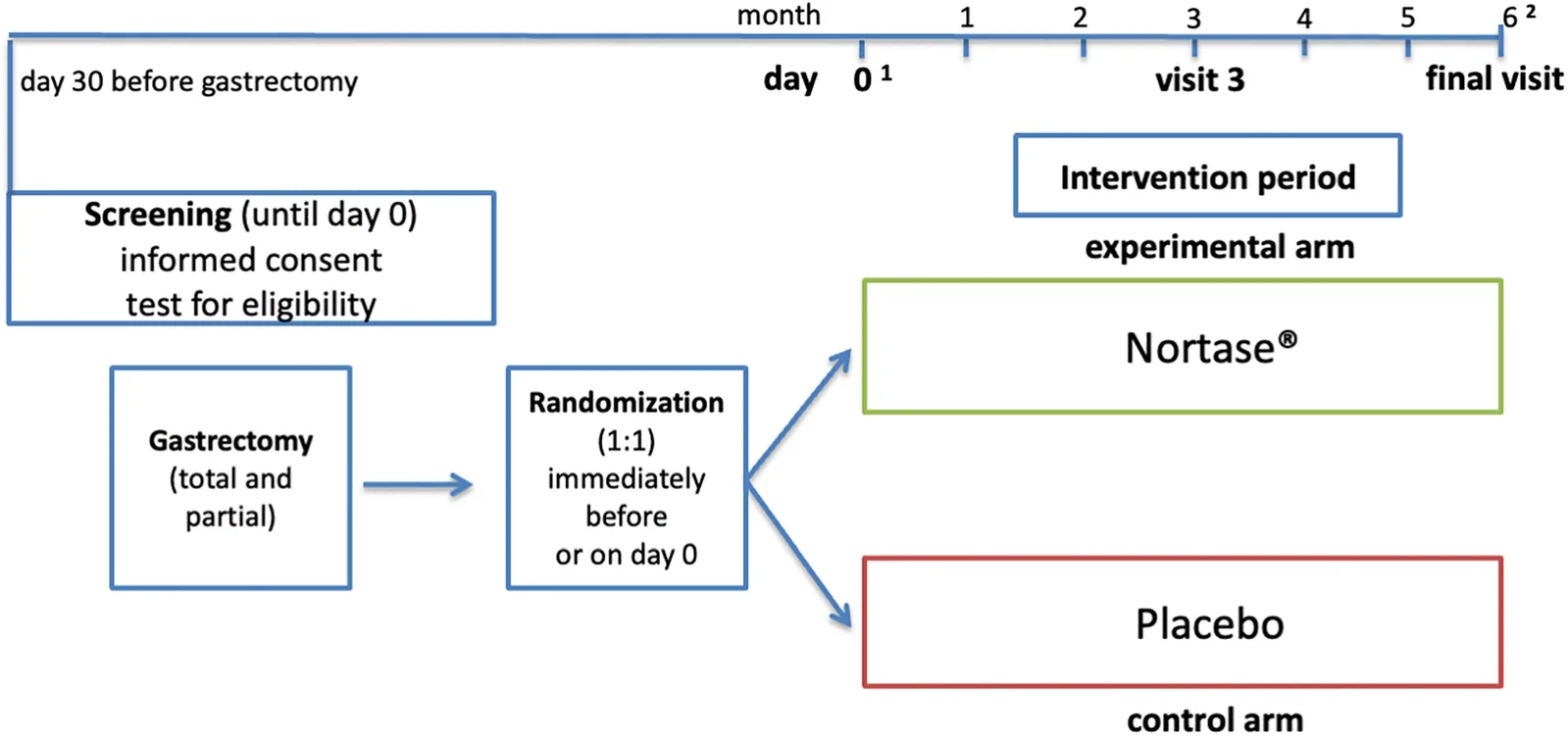
Timeline of an individual participant. ^1^ ideally with the begin of oral nutrition, max. 3 days after begin of oral nutrition; ^2^ following, every 6 months, requests (by telephone) on the survival status until the end of the overall trial.

Figures 1 and 2 depict the assessments and procedural workflow as well as the schedule for an individual participating in the trial.

Patients will be recruited only at the trial sites, which are all specialized in gastrointestinal disorders, nutritional medicine and care of patients after operations on the gastrointestinal tract.

First, a screening visit not more than 30 days before gastrectomy takes place at which inclusion and exclusion criteria will be carefully checked, QOL is assessed and blood samples will be taken. Additionally, eating habits are screened at initial visit as stated above.

At day 0 (= visit 1) randomization will be performed, and medication will be started with the beginning of oral nutrition, including distribution of half of the trial medication. Survival status is collected in all subsequent visits.

Three weeks after day 0 a telephone visit is conducted (= visit 2). Assessment of QOL and eating behavior is repeated. Putative adverse events are ascertained and classified (see section below).

Three months after randomization (= visit 3) the same assessments as at visit 1 are performed again and the remaining trial medication is handed out.

Six months after randomization (= visit 4) examinations are repeated. Subsequently, participants will be screened for survival every six months via telephone contact until trial termination. To assess compliance, the number of returned bottles of medication will be documented. Moreover, patients are asked to keep a medication diary, which is reviewed at study visits and consulted upon asking them to assess their own adherence to medication.

Patients may withdraw their consent to participate at any time without giving reasons. Information as to when and why a patient was registered or randomized and when he withdrew consent will be retained in the documentation.

Trial therapy must be terminated prematurely in case of an adverse event occurred to a patient that would lead to an unacceptable risk for this patient if treatment was not discontinued. Beyond that, the trial must be terminated if the worsening of a patient (who is not responding to treatment) would contraindicate the treatment with trial therapy transiently or permanently.

### Sample size and recruitment

A total of 188 patients will be randomized to reach a power of 80% with a two-sided significance level of 5% and accounting for 10% loss to follow-up.

The minimal clinically important difference for QOL sub-scales from the EORTC-QLQ-C30 is often taken to be half of the standard deviation (SD) or 10 points across a range of cancers [24–26]. Avery et al. report on patients similar to those in this trial where QOL for the physical function is 80–85 points at baseline with an SD of about 23 [27]. To detect a minimal clinical important difference of 10 with an SD of 23 using a two-sided t-test with 80% power at the 5% significance level, 84 patients per group need to be analyzed.

The anticipated drop-out rate is low, e.g., after one year, there were no dropouts amongst 43 randomized gastrectomy patients in Catarci et al [14]. Moreover, mortality can be expected to be under 5% at 6 months in patients with curative gastrectomy [28–30]. However, we allow for 10% missing values in the questionnaire used for the primary endpoint. Patients who terminate use of the medication/placebo, will be retained in the final primary intention to treat analysis. Patients who die before reaching the primary endpoint will be replaced by newly randomized patients and if data for the primary endpoint are missing from more than 10% of the patients, additional patients may be recruited.

Note that the sample size calculation assumes that the intraclass correlation coefficient (ICC) between trial sites is negligible, which has little impact as long as ICC < 0.05 [31]. Particularly since relevant covariates are included in the analysis model, this assumption is expected to hold well.

Recruitment will proceed until the pre-determined sample size is reached. If recruitment is considerably slower than anticipated, then a pooled estimate of variance and of drop-out/missing data can be used to see if a power of 80% can be attained with a smaller sample size. If so, then recruitment can be terminated after reaching the adjusted sample size, which will be considered a substantial amendment.

The first participant was randomized on January 24 2024 and recruitment is ongoing. Last patient out is expected in February 2028. Accordingly, data collection and final analyses are presumed to be completed thereafter, with results anticipated to become available during the course of 2028.

### Allocation and blinding

This is a double-blind trial in which the biometrician responsible for data analysis is also blinded. Trial medication and placebo will be manufactured and labelled with a completely non-informative three-character code by Repha using a list provided by Clinical Trial Centre Leipzig.

Patients will be randomized in a 1:1 ratio after gastrectomy and before beginning with oral intake of food. We randomize after surgery to exclude allocating patients with intra-operative mortality and severe complications. Patients will be randomized using variable length block randomization, stratified by trial site. It is not logistically feasible in this trial to account for other stratification variables, but relevant risk variables will be taken into account in the analysis methods.

Information regarding the treatment arm of a patient will be kept secret in a sealed and light-proof envelope to be opened only in emergencies or in the case of serious adverse events that require immediate medical intervention.

If an investigator, site personnel performing assessments, or patient is unblinded, the patient remains in the trial unless exclusion is deemed necessary for safety reasons by the coordinating investigator.

### Adverse events

Safety analyses will be conducted for all patients that received at least one dose of placebo/medication. Patients will be analyzed according to the treatment received.

Although pancreas enzyme administration is well tolerated, known side effects of enzyme replacement therapy are monitored according to the SmPC. In rare cases nonspecific concomitant symptoms such as diarrhea, nausea, obstipation, and upper abdominal discomfort, as well as allergic respiratory and skin reactions have been observed following occupational sensitization with mold enzymes.

Adverse events (AE) and severe adverse events (SAE) will be documented on special forms from start of therapy (day 0) until the final visit (visit 4) according to the detailed guidance on the collection, verification and presentation of adverse event/reaction reports arising from clinical trials on medicinal products for human use.

All abnormal physical and/or laboratory results which are considered to be clinically relevant by the investigator should be recorded as AEs. If an abnormal laboratory result meets any of the criteria for a SAE, this must also be reported on the SAE form.

Adverse Events are classified by their seriousness, intensity/severity and relationship to the individual medical procedure.

The following exceptions from the documentation of (serious) adverse events are made: (a) bleeding as a result of gastrectomy, (b) wound healing disorders as a result of gastrectomy, (c) postoperative infections, (d) events resulting from a re-gastrectomy including post-treatment, (e) events resulting from radiation or chemotherapy.

### Data processing and management

In the context of a database for electronic data capture only, the Case Report Form (CRF) will be designed by the Clinical Trial Centre Leipzig in cooperation with the sponsor’s authorized representative and provided as electronic form (eCRF). In order to facilitate the documentation as per protocol in case of malfunction of the electronic system or any of its components, a paper version of the CRF will be provided in the Investigator Site File (ISF). The data on this paper version will be transferred to the eCRF as soon as the electronic system is available again.

An eCRF will be provided for each patient. The patient will be identified as per patient-ID only. The eCRF must be completed shortly after each trial visit according to ICH Guideline E6 chapter 4.9.1 and to enable central monitoring of the trial data.

Access to the database will be limited to authorized staff only. Authorization is granted by the site’s investigator using the trial specific staff signature and delegation log. Based on the staff signature and delegation log access to the eCRF will be granted by the responsible staff at the Clinical Trial Centre Leipzig.

Authorized staff members on site will be able to enter and update data as well as finalize data by electronic signature during the conduct of the trial according to a trial specific concept for documentation.

The information entered into the eCRF by the investigator or an authorized member of the trial team is systematically checked for completeness, consistency and plausibility by routines such that discrepancies can be dealt with at data entry.

During the whole course of the trial, a backup of the data is made on a daily basis. Unauthorized access to patient data is prevented by the access concept of the trial database, which is based on a strict hierarchy and role concept. Any change of data (e.g., when data is changed in the database during query management) is recorded automatically within the database.

At the end of the trial, once the database has been declared complete and accurate, the database will be locked. Thereafter, any changes to the database are possible only by joint written agreement between sponsor, authorized representative/coordinating investigator, biometrician and data manager.

All relevant trial documentation, i.e., the trial master file, the electronically stored data, the original CRFs and the final report will be stored for at least 25 years at the university archive Leipzig after the regular or premature end of the trial.

At the investigating sites, the investigators’ files, patient identification lists, signed written consent forms, copies of all CRFs and the patients’ files will be stored for at least 25 years after the regular or premature end of the trial. Local rules or other legal requirements are to be met if they require longer periods of archiving.

### Monitoring

A data monitoring and safety board is not foreseen in this trial, since safety issues related to the medicinal product are not expected as it is not absorbed and all adverse events will be monitored closely and in a timely matter. Moreover, data quality will be reviewed by the trial team in monthly reports as part of the central and statistical monitoring in accordance with ICH E6 (R2). The Clinical Trial Centre Leipzig will be responsible for one-site monitoring. Initiation, regular and close-out visits will be performed in all trial sites. A risk-based monitoring strategy will be implemented, as required by ICH E6 (R2, Chapter 5.0).

### Statistical analysis

The primary analysis will be based on all randomized patients alive at 6 months and using the randomization arm they were allocated to, irrespective of compliance. As noted above, patients will only be recruited for this trial if the gastrectomy is curative and their prognosis is fairly good, meaning that mortality within 6 months will be very rare. If a patient should die before reaching the primary endpoint, this patient will be excluded from the analysis since the gastric cancer is expected to dominate QOL as opposed to the gastrectomy, meaning the therapy is not expected to be particularly effective, the data are uninformative, and the imputation techniques used otherwise may well induce bias. This is a principal stratum analysis for survivors based on the reasonable assumption that the therapy does not affect survival.

A linear mixed repeated measures model will be used to analyze the primary endpoint. The dependent variable is the physical sub-score of the EORTC-QLQ-C30 at 3 and 6 months and its value at baseline as well as (a) sex, (b) partial vs total gastrectomy, (c) previous/planned chemotherapy vs none and (d) pre-operative BMI ≥ 25 kg/m^2^ and (e) the time point 3 or 6 months as a categorical variable. These variables were chosen based on risk factors for post-gastrectomy weight loss [32]. Trial site will be treated as a random effect. Missing data are accounted for implicitly by the mixed model in the sense that all patients who provide data at at least one point in time are retained in the model and contribute to the estimates at each point in time. Note that for a mixed model and multiple imputation method using the same analysis model, estimates will be similar asymptotically and the mixed model will be more efficient [33]. The contrast at 6 months between the arms constitutes the primary endpoint and the estimate will be accompanied by a 95% Wald confidence interval and the p-value dual to it. A linear model with complete data and without consideration of the trial site will serve as a sensitivity analysis. A second sensitivity analysis will repeat the primary analysis after including patients who have died and setting their missing data to the overall lowest observed value for the endpoint (i.e., the lowest value among all patients). A third sensitivity analysis will repeat the primary one after excluding patients from sites with very few patients, since their inclusion could affect the estimates of the random term unduly. Descriptive statistics will be used for the distribution of the cumulative dose of medication, separated by arm. The association with the primary endpoint will also be provided descriptively. The primary analysis does not include dose, since the primary goal of the trial is to demonstrate effectiveness and not efficacy (dose modifications are routine and thus belong to an assessment of effectiveness). Moreover, a dependence on dosage would be extremely difficult to interpret, since higher doses may be the causal result of patient dissatisfaction/discomfort, which would in turn be reflected in the primary endpoint.

Secondary endpoints for continuous data will be analyzed along the same lines as the primary analysis, but without the corresponding sensitivity analysis. For binary variables (e.g., 10% weight loss at six months) an analogous generalized linear mixed model with a logit link function will be used and a profiling method to construct confidence intervals. The p-values will be complementary to the confidence intervals. Mortality will be presented graphically with a Kaplan-Meier curve. Since the intervention is not intended or expected to affect mortality and since the trial is underpowered for this endpoint, no formal statistical test of mortality is foreseen. However, a Cox regression using the same covariates as with the primary endpoint will be performed to estimate the hazard ratio and provide a 95% confidence interval for it, after ensuring that the proportional hazards assumption is reasonable. Note that for this descriptive analysis, all randomized patients will be included.

A priori subgroup analyses are not foreseen and no interim analysis is planned.

### Publication policy

Authorship will follow the criteria for authorship developed by the International Committee of Medical Journal Editors, including those that distinguish authors from other contributors. Each site that contributes at least 3 patients to the full analysis set and has complete documentation for these patients can list a single author. Each site that contributes at least 8 patients to the full analysis set and has complete documentation for these patients can list two authors. The first and senior authorships are reserved for the team of the coordinating investigator and the order of authors from recruiting sites depends on the number of patients recruited from the respective site.

## Discussion

Malnourishment is a common problem in patients after partial or total gastrectomy. As revealed in many studies, alterations in physiological processes after gastrointestinal surgery finally may result in maldigestion and malabsorption, accompanied by weight loss, discomfort and impaired QOL. EPI emerged as a key pathological mechanism promoting these symptom [8]. In a recent meta-analysis, incidence of EPI varied from 23% after bariatric metabolic surgery, up to 47% after partial and 66% after total gastrectomy [10].

Few studies highlighted the role of pancreas enzyme supplementation to improve gastrectomy related symptoms and malnutrition, respectively. Improvements in clinical symptoms among pancreas enzyme therapy such as steatorrhea or abdominal pain showed inconsistent results, while some evidence suggest enhanced QOL in this cohort [11,13,14]. As discussed in the Introduction, however, previous trials showed a lack of generalizability, small sample size and had to deal with methodological restrictions, yielding a demand for well-designed, controlled large trials [8]. So far, recommendations concerning pancreas enzyme supplementation remained heterogeneous and barely evidence based.

Therefore, we designed PANEM as a prospective randomized placebo-controlled trial to ascertain and further evaluate the role of pancreas enzyme substitution over six months in patients after gastrectomy. QOL after these six months of administration will be regarded as primary objective while we address weight loss, nutritional status and other disease-related comorbidities and laboratory measurements as secondary objectives.

Statistical analysis was carefully performed to ensure adequate power. Based on previous findings we seek to include 188 patients in total, randomized 1:1 to either placebo or verum, yielding in a sufficient number of enrolled patients to examine superiority of pancreas enzyme substitution. Beyond that, multi-centered recruitment promotes generalizability of our trial.

Summing up, PANEM will hopefully help to improve treatment of gastrectomy associated disorders and to prevent discomfort and weight loss in these patients, ideally resulting in an evident recommendation in favor or against pancreas enzyme therapy in this cohort.

## Supporting information

S1 Checklist SPIRIT Checklist. (PDF)

S2 Protocol Study protocol of the PANEM trial. (PDF)

## Abbreviations

AE: adverse event
BMI: body mass index
CRF: case report form
eCRF: electronic case report form
EORTC QLQ-C30: EORTC Core Quality of Life Questionnaire
EPI: exocrine pancreas insufficiency
FIP: Fédération Internationale Pharmaceutique
ISF: Investigator Site File
QOL: quality of life
SAE: severe adverse event
SD: standard deviation
SmPC: Summary of Product Characteristics
UICC: Union for International Cancer Control.
