# Supplementary material for: Protocol of the PANEM trial: A randomized, phase III, parallel-arm, placebo-controlled, double-blind, multi-center superiority trial comparing PANcreatic EnzyMes versus placebo in adults after gastrectomy
Source: PLoS One. 2026 Sep 22;21(9):e0358177. doi: 10.1371/journal.pone.0358177 (PMC13596798; doi:10.1371/journal.pone.0358177)
Supplement: Supplementary file 2 [file pone.0358177.s002.pdf]

Trial Protocol

The **<u>Pan</u>**creatic **<u>E</u>**nzy**<u>m</u>**es after Gastrectomy Trial

PANEM

Sponsor Representative	Prof. Dr. med. habil. Albrecht Hoffmeister<br>Klinik für Onkologie, Gastroenterologie, Hepatologie und Pneumologie<br>Universitätsklinikum Leipzig<br>Liebigstr. 20, 04103 Leipzig
Sponsor	Universität Leipzig<br>Ritterstraße 26, 04109 Leipzig

Date:	19.04.2024
Version:	final 3.0
EU Trial Number:	2022-501944-14-00
ClinicalTrials.gov - no.:	NCT06058442

Table of Contents

PAGE

GENERAL INFORMATION........5

Responsible Parties........5
Synopsis........7
Synopse (German version)........9
Schedule of Assessments and Procedures........11
Flow Chart........12

1 CONFIRMATION OF COMPLIANCE........13

2 RATIONALE........14

2.1 Medical Background........14
2.2 Rationale........14
2.3 Evidence........14
2.4 Risk-Benefit Consideration........15

3 OBJECTIVES........16

3.1 Primary Objective........16
3.2 Secondary Objectives........16

4 TRIAL DESIGN AND DESCRIPTION........17

4.1 Trial Design........17
4.2 Low intervention clinical trial........17
4.3 Patient Involvement........17
4.4 Trial Sites and Number of Trial Subjects........17
4.5 Expected Duration of Trial........17
4.6 Premature Termination of the Trial........18

5 TRIAL SUBJECTS........19

5.1 Inclusion Criteria........19
5.2 Exclusion Criteria........19
5.3 Justification for the Inclusion of vulnerable populations........21
5.4 Participation in more than one Clinical Trial........21
5.5 Rationale for Sex and Age Distribution........21

6 INVESTIGATIONAL PRODUCT........22

6.1 Trial Drugs........22
6.2 Packaging and Labelling of the Trial Drug........22
6.3 Storage and Handling Requirements........23
6.4 Drug Accountability........23
6.5 Administration of Trial Drug........24
6.6 Blinding and Unblinding........25

7 INDIVIDUAL TRIAL PROCEDURES........27

7.1 Patient Information and Informed Consent........27

7.2 Enrolment in the Trial 28
7.3 Screening (max. 30 days before gastrectomy until visit 1) 28
7.4 Description of the Treatment Procedures 29
7.5 Description of Trial Visits 30
7.6 Premature Termination of the Therapy or Follow-up 31

8 ADVERSE EVENTS (AE/SAE) 33

8.1 Adverse Events (AE) 33
8.2 Safety Analysis 34
8.3 Concomitant Diseases 34
8.4 Serious Adverse Events/Serious Adverse Reactions (SAE/SAR) 34
8.5 Periodic Reports 35
8.6 Suspected Unexpected Serious Adverse Reactions (SUSAR) 36
8.7 Follow up of Adverse Reactions 37
8.8 Other Safety Relevant Issues 37
8.9 Therapeutic Procedures 37
8.10 Dealing with Pregnancy 37

9 BIOMETRY 39

9.1 Biometrical Aspects of the Trial Design 39
9.2 End Points 39
9.3 Statistical Description of the trial hypothesis 40
9.4 Sample Size Discussion 41
9.5 Statistical Methods 42
9.6 Statistical Monitoring 43
9.7 Interim Analysis 44
9.8 Final Analysis 44

10 DOCUMENTATION 44

10.1 General information and Access Rights 44
10.2 Patient File and Source Data 45
10.3 Data Management 45
10.4 Archiving 46

11 SUPERVISION OF THE CLINICAL TRIAL 46

11.1 Sponsor Statement regarding supervision of trial and access to source data 46
11.2 Monitoring 46
11.3 Audits 47
11.4 Inspections 47

12 DATA PROTECTION 47

12.1 Data protection and Confidentiality 47
12.2 Anonymisation of Data after the end of Archiving 48

13 ADMINISTRATIVE AGREEMENTS 48

13.1 Adherence to the Protocol 49
13.2 Serious Breaches 49
13.3 Funding and Insurance 49
13.4 Publication Policy and Registration 50

13.5 Data Sharing Statement....50
14 PROTOCOL SIGNATURES....52
15 PROTOCOL AGREEMENT....53
16 APPENDIX....54
16.1 Classification of Adverse Events....54
16.2 Acronyms....56
16.3 Labelling....58
16.4 Template trial protocol....58
17 REFERENCES....59

GENERAL INFORMATION[1]

Responsible Parties

Sponsor	**Leipzig University**<br>Ritterstr. 26, 04109 Leipzig
Sponsor Representative	Prof. Dr. med. habil. Albrecht Hoffmeister<br>Klinik und Poliklinik für Onkologie, Gastroenterologie, Hepatologie und Pneumologie,<br>Universitätsklinikum Leipzig<br>Liebigstraße 20, 04103 Leipzig<br>Tel: ++49 (0)341/9712240; Fax: ++49 (0)341/9712209<br>Albrecht.Hoffmeister@medizin.uni-leipzig.de
Deputy of Sponsor Representative	Klinik und Poliklinik für Onkologie, Gastroenterologie, Hepatologie und Pneumologie,<br>Universitätsklinikum Leipzig
Project Management	Zentrum für klinische Studien Leipzig<br>Universität Leipzig<br>Medizinische Fakultät
Pharmacovigilance	Zentrum für klinische Studien Leipzig<br>Universität Leipzig<br>Medizinische Fakultät
Data Management	Zentrum für klinische Studien Leipzig<br>Universität Leipzig<br>Medizinische Fakultät
Biometry	Zentrum für klinische Studien Leipzig<br>Universität Leipzig<br>Medizinische Fakultät

[1] The contents of this trial protocol refer to women and men equally. However, for reasons of better readability, the male form is chosen for all personal designations. The female form is always taken into account. An exception are those contents solely referring to women.

Monitoring	Zentrum für klinische Studien Leipzig<br>Universität Leipzig<br>Medizinische Fakultät
Funding	Repha GmbH Biologische Arzneimittel

Synopsis

Title of the trial	The Pancreatic Enzymes after Gastrectomy Trial
Acronym	PANEM
Indication	Patients after gastrectomy (total and partial)
Primary objective of the trial/ primary endpoint	**Primary objective:**<br>Can enzyme supplementation with every meal after gastrectomy lead to improved disease specific quality of life at 6 months after gastrectomy?<br>**Primary endpoint:**<br>Disease specific quality of life (QOL) at 6 months according to the physical sub-score of the EORTC-QLQ-C30
Secondary objectives of the trial/secondary endpoints	**Secondary objectives:**<br>Can enzyme supplementation with every meal after gastrectomy lead to less weight loss and better nutritional status after gastrectomy?<br>**Secondary endpoints:**<br>• Sub-scales of QOL at three and six months (GILQI, EORTC-QLQ-C30 / STO22)<br>• proportion of patients who receive supplemental nutrition after the run-in period of 3 weeks or have > 10% weight loss at six months.<br>• % weight change at six months<br>• blood parameters at six months (serum haemoglobin, total protein, albumin, glucose, HbA1c, cholesterol, vitamins A, B12, D, E and K)<br>• **Optional**, all patients at selected trial sites: faecal elastase-1 test (FE-1) at six months<br>• mortality: Patients will be contacted at regular intervals to acquire mortality data including date of death until last patient-out
Trial design	Randomized, phase 3, parallel arm, placebo-controlled, double blinded, multi-centred, superiority trial
Trial population	**Key inclusion criteria**<br>• Gastrectomy (total and partial)<br>- at least ≥ 50 % of the stomach must be resected<br>• Age 18 or older<br>**Key exclusion criteria**<br>• Indication for pancreas enzyme therapy<br>• Gastrectomy with palliative intention<br>• UICC (Union for International Cancer Control) Stage IV gastric malignancy unless surgery is planned according to new guidelines in oligometastatic disease<br>• Malnutrition of other aetiology

	• Life expectancy < 12 months<br>• Known lactose intolerance<br>• Known hereditary galactose intolerance<br>• Patients on alpha-glucosidase inhibitors (AGIs)<br>• Acute pancreatitis<br>• Acute episode of chronic pancreatitis<br>• Known hypersensitivity to moulds (mould allergy) or any other ingredient of NORTASE®<br><br>Complete list of criteria: see section 5.1 and 5.2 in the protocol
Sample size	To be randomized to trial: n=188<br>To be analysed: n= 188 (ITT) 168 (per protocol)
Therapy	**Intervention:**<br>Patients should take 10-15 capsules of NORTASE® **or** placebo per day<br>**Duration of intervention:** 6 months
Biometry	**Primary endpoint:** Linear mixed model with the physical sub-score of the EORTC-QLQ-C30 at 6 months as dependent variable and its value at baseline as well as (a) sex, (b) partial vs total gastrectomy, (c) previous/planned chemotherapy vs none and (d) pre-operative BMI ≥ 25 kg/m². Trial site will be treated as a random effect. **Secondary endpoints:** Continuous data will be analysed along the same lines as the primary analysis. For binary variables (e.g. 10% weight loss at six months) an analogous generalized linear mixed model with a logit link function will be used and a profiling method to construct confidence intervals. The p-values will be complementary to the confidence intervals. Mortality will be presented graphically with a Kaplan-Meier curve and an estimate of the hazard ratio with 95% CI taken from a Cox regression using the same covariates as with the primary endpoint.
Trial Duration	**Patient related:**<br>6 months of treatment<br>**All** patients will be followed until the end of the entire trial (LPLV = last patient last visit). Survival visits will take place every 6 months until LPLV.<br><br>**Trial related:**<br>Recruitment period: 36 months<br>First patient in to last patient out: 42 months<br>Duration of the entire trial, including preparation and analysis: 54 months

Synopse (German version)

Titel der Studie	Pankreatische Enzyme nach Gastrektomie
Kurzbezeichnung der Studie	PANEM
Indikation	Patienten nach (totaler) Gastrektomie oder (partieller) Magenresektion
Primäres Ziel der Studie/ primärer Endpunkt	**Primäres Ziel:**<br>Kann nach einer Gastrektomie die Supplementierung von Enzymen zu jeder Mahlzeit zu einer Verbesserung der krankheitsspezifischen Lebensqualität nach 6 Monaten führen?<br>**Primärer Endpunkt:**<br>Krankheitsspezifische Lebensqualität (QOL) nach 6 Monaten gemäß dem physischen Sub-Score des EORTC-QLQ-C30
Sekundäre Ziele der Studie/ sekundäre Endpunkte	**Sekundäres Ziel:**<br>Kann eine Enzym-Supplementierung zu jeder Mahlzeit nach einer Gastrektomie zu einem geringeren Gewichtsverlust und einem besseren Ernährungszustand führen?<br>**Sekundäre Endpunkte:**<br>• Sub-Skalen der Lebensqualität nach drei und sechs Monaten (GILQI, EORTC-QLQ-C30 / STO22)<br>• Anteil der Patienten, die nach der Einführungsphase von 3 Wochen eine Zusatznahrung benötigen oder nach sechs Monaten einen Gewichtsverlust von > 10 % aufweisen.<br>• prozentuale Gewichtsveränderung nach sechs Monaten<br>• Blutparameter nach sechs Monaten (Serumhämoglobin, Gesamtprotein, Albumin, Glukose, HbA1c, Cholesterin, Vitamine A, B12, D, E und K)<br>• **Fakultativ:** alle Patienten an ausgewählten Prüfzentren: Fäkaler Elastase-1-Test (FE-1) nach sechs Monaten<br>• Sterblichkeit: Die Patienten werden in regelmäßigen Abständen kontaktiert, um Mortalitätsdaten (inkl. Todesdatum) zu erfassen, bis der letzte Patient die Studie beendet hat
Studiendesign	Randomisierte, parallel-armige, Placebo-kontrollierte, doppel-blinde, multizentrische Überlegenheitsstudie der Phase 3
Studienpopulation	**Wichtigste Einschlusskriterien**<br>• Gastrektomie oder (partielle) Magenresektion<br>- ≥ 50 % des Magens müssen reseziert werden<br>• Alter 18 Jahre oder älter<br><br>**Wichtigste Ausschlusskriterien**<br>• Indikation für eine Pankreasenzymtherapie<br>• Gastrektomie mit palliativer Intention<br>• UICC (Union for International Cancer Control) Stadium IV eines malignen Magenkarzinoms; ausgenommen die Operation wird gemäß den neuen Leitlinien für oligometastatische Erkrankungen geplant

	• Mangelernährung anderer Ätiologie<br>• Lebenserwartung < 12 Monate<br>• Bekannte Laktoseintoleranz<br>• Bekannte hereditäre Galaktoseintoleranz<br>• Patienten, die Alpha-Glucosidase-Inhibitoren (AGIs) einnehmen<br>• Akute Pankreatitis<br>• Akute Episode einer chronischen Pankreatitis<br>• Bekannte Überempfindlichkeit gegen Schimmelpilze (Schimmelpilzallergie) oder einen anderen Inhaltsstoff von NORTASE®<br><br>Die vollständige Liste der Kriterien: siehe Abschnitt 5.1 und 5.2 des Protokolls
Patientenzahl	Zu randomisieren: n=188<br>Zu analysieren: n=188 (ITT), 168 (per protocol)
Therapie	**Intervention:**<br>Patienten sollen 10-15 Kapseln NORTASE® **oder** Placebo täglich einnehmen<br>**Dauer der Intervention**: 6 Monate
Biometrie	**Primärer Endpunkt:** Lineares gemischtes Modell mit der physischen Sub-Score des EORTC-QLQ-C30 zu 6 Monaten als abhängige Variable und der Wert zu Baseline sowie (a) Geschlecht, (b) totale vs partielle Resektion, (c) vorherige bze. geplannte Chemotherapie vs keine und (d) präoperatives BMI ≥ 25 kg/m². Zentrum wird als „random Term“ behandelt. **Sekundäre Endpunkte:** Stetige Variablen warden analog zum primären Endpunkt ausgewertet. Für binäre Variablen (z. B. 10 % Gewichtsverlust nach sechs Monaten) wird ein generalisiertes lineares gemischtes Modell mit logit Link-Funktion aber sonst analog zum primären Endpunkt verwendet. Profiling-Methoden werden zur Konstruktion des Konfidenzintervalls verwendet und die p-Werte sind complementär dazu. Mortalität wird mit einer Kaplan-Meier Kurve dargestellt und das hazard ratio mit Konfidenzintervall über eine Cox-Regression mit denselben Variablen wie beim primären Endpunkt bestimmt.
Zeitplan	**Patienten-bezogen:**<br>6 Monate Behandlung<br>**Alle** Patienten werden bis zum Ende der gesamten Studie beobachtet (LPLV = last patient last visit). Alle 6 Monate bis zu LPLV erfolgt eine Abfrage zum Überlebensstatus.<br>**Studien-bezogen:**<br>Rekrutierungszeitraum: 36 Monate<br>*First patient in* bis *last patient out*: 42 Monate<br>Dauer der gesamten Studie, einschließlich Vorbereitung und Analyse: 54 Monate

Schedule of Assessments and Procedures

Visit / Time	Screening (Scr) max. 30 days before gastrectomy until day 0	Visit 1 (V1) Day 0[3] Start of medication	Visit 2 (V2)[2] Week 3 +/- 1 week	Visit 3 (V3) Month 3 +/- 2 weeks	Final visit (V4) Month 6 +/- 4 weeks	Survival visit(s) (SV) every 6 months after month 6
Informed consent	●					
Eligibility criteria	●					
Randomisation[4]		●				
Medical/Medication History	●					
Demographics	●					
Assessment of vital signs [5]	●	(●)[6]		●	●	
Blood analysis[7]	●	(●)[6]		●	●	
Eating habits and nutrition	●		●	●	●	
Quality of life questionnaires	●		●	●	●	
Adverse Events			●	●	●	
Concomitant medication	●	(●)[6]	●	●	●	
Distribution / first intake of trial medication at V1		●		●		
Drug Accountability				●	●	
Survival status			●	●	●	●
Faecal elastase-1 test[8]	●				●	

---

[2] if necessary, only by phone
[3] ideally with the begin of oral nutrition, max. 3 days after begin of oral nutrition
[4] after gastrectomy, immediately before or at day 0
[5] arterial blood pressure, heart rate, temperature, body weight
[6] if screening visit occurs on day 0 (visit 1), these examinations are only performed once
[7] serum haemoglobin, total protein, albumin at visits Scr, V1, V3 and V4,
glucose, HbA1c, cholesterol, vitamins A, B12, D, E and K only needs to be measured once, either for visit Scr **OR** V1 **AND** V3 and V4
pregnancy test only at screening – in blood or urine
[8] optional, all patients at selected trial sites

Flow Chart

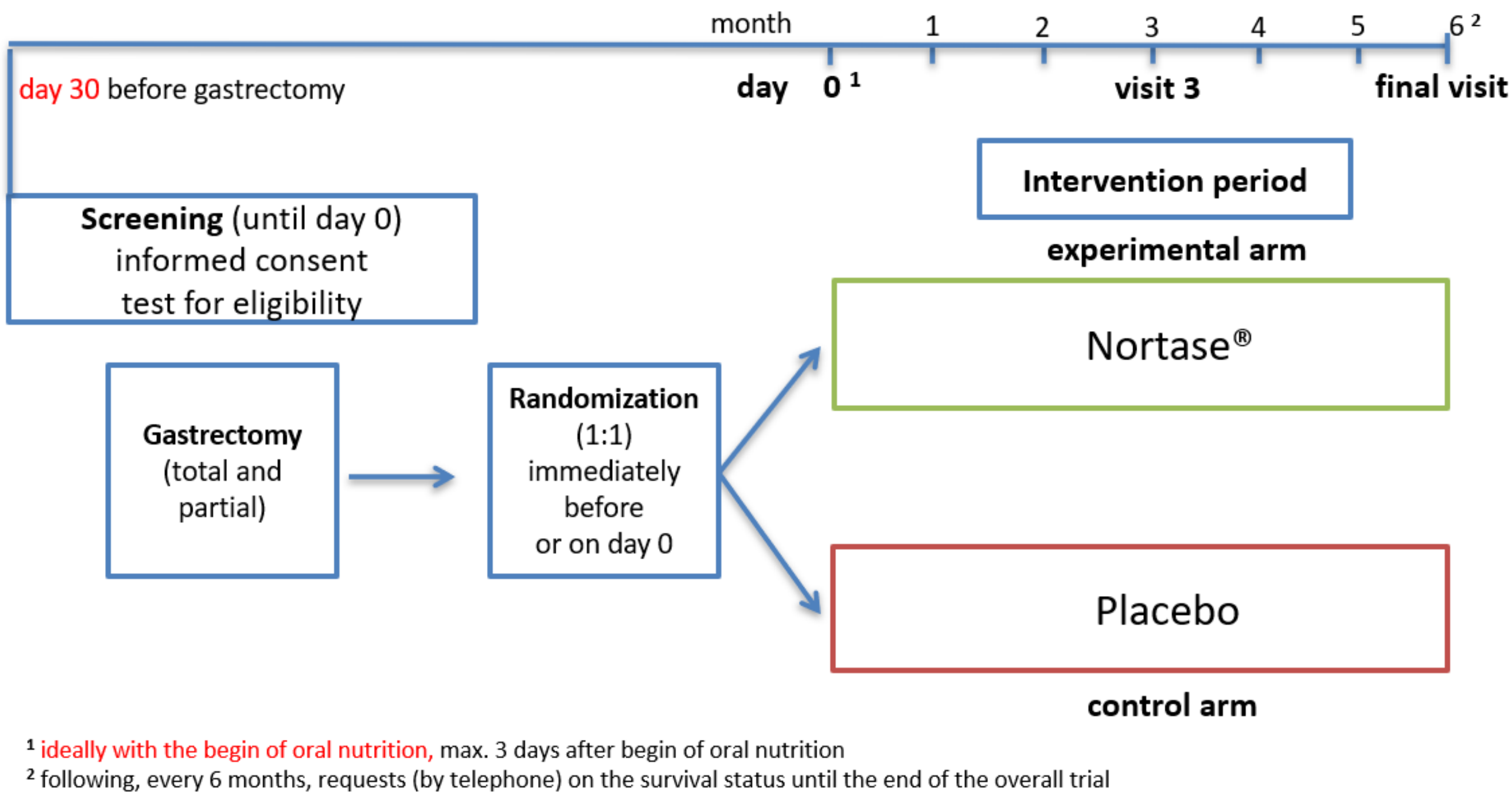
month
1
2
3
4
5
6 ²
day 30 before gastrectomy
day 0 ¹
visit 3
final visit
Intervention period
experimental arm
Screening (until day 0)
informed consent
test for eligibility
Nortase®
Gastrectomy
(total and
partial)
Randomization
(1:1)
immediately
before
or on day 0
Placebo
control arm
¹ ideally with the begin of oral nutrition, max. 3 days after begin of oral nutrition
² following, every 6 months, requests (by telephone) on the survival status until the end of the overall trial

1 CONFIRMATION OF COMPLIANCE

All persons participating in the conduct of the trial (sponsor, authorised representative of the sponsor, investigators, etc.) commit themselves to conduct the trial according to this protocol, the Declaration of Helsinki of the WMA (in its current version), as well as the EU CTR 536/2014 and the ICH guideline for Good Clinical Practice (GCP) ICH E6(R2) (EMA/*CHMP/ICH*/135/1995) issued in June 2017.[1]

2 RATIONALE

2.1 Medical Background

The most common patients of the trial will be with gastric cancer because gastrectomies for other indications are very rare. Gastric cancer is the fourth (men) or fifth (women) most prevalent form of cancer and accounts for 10% of deaths due to cancer worldwide.[2] Guidelines recommend gastrectomy for the majority of patients,[2] often with curative intent, and improvements in prognosis have been seen over the last decades.[3] Despite these advancements, malnourishment is common after gastrectomy and is associated with post-operative mortality and morbidity.[4]

2.2 Rationale

The anatomical changes due to the gastrectomy lead to physiological changes: food is exposed to the digestive enzymes produced by the pancreas at a later point within the gut, meaning that the digestive process is delayed. The net result could be maldigestion and malabsorption leading to malnutrition with weight loss, steatorrhoea and discomfort. By taking digestive (for example pancreatic) enzymes together with meals, it may be possible to bring about improvements in digestion and thus lessening of these problems. Overall, this is expected to result in an improved quality of life.

Digestive enzymes (NORTASE®) will be administered as recommended by the manufacturer (i.e. in capsules, taken orally with a dosage of 10–15 capsules per day, i.e. 2–3 per meal, with five small meals per day in this particular patient population). Since the enzyme mixture works locally in contact with food, only oral administration is possible. Since there are no guideline recommendations pertaining to enzyme supplementation in these patients, a placebo control group is deemed ethical and maximally informative. As demonstrated below (see 2.3) there is currently insufficient evidence regarding the potential benefit of enzyme supplementation and this trial will answer the pertinent questions (see 9.4).

The effectiveness of digestive enzymes regarding improved metabolism and clinical consequences for digestion and weight will be considered as secondary endpoints. The primary goal of this pragmatic and patient-oriented trial is to measure the potential benefit of the intervention on quality of life.

2.3 Evidence

A very small, older, double-blinded crossover trial (n=15) compared the supplementation of pancreatic enzymes with placebo in gastrectomy patients over only 7 days to assess abdominal symptoms and stool properties and found improvements in steatorrhoea and stool consistency.[5] The same group performed a second double-blind RCT about ten years later (n=52) with 14 days of treatment and similar endpoints, but did not find meaningful or significant effects, though improvement in quality of life was detected.[6] An open-label RCT comparing “normal diet” to supplementation of pancreatic enzymes at meals with a treatment and observation time of 12 months found no effect on BMI, but some indication for improvements in quality of life and nutritional status.[7] The main limitation of this last trial is the open label design and its low sample size (n=43), which was based on the extremely optimistic expectation that BMI in the intervention group would be 20% higher than in the control group.

Reviews conclude that effects of pancreatic enzymes in gastrectomy patients appear to be small, but that evidence is lacking, and that the RCTs performed were of “small sample size and low quality”.[8]

Studies with supplementation of other digestive enzymes after gastrectomy are not available.

2.4 Risk-Benefit Consideration

The physiological considerations and the scant evidence noted above suggest that enzyme supplementation can be expected to benefit patients regarding quality of life, particularly pertaining to gastrointestinal issues, and perhaps regarding unwanted weight loss. The known side-effects of the enzyme supplementation NORTASE® are rare (less frequent then 1/1000) and unspecific suggesting a risk-benefit ratio clearly in favour of the potential benefits. A double-blind design is ethically justifiable since patients do not receive enzyme supplementation as part of standard care and the physician can take treatment decisions without knowing if the patient is receiving verum or placebo. The trial subjects (inclusion/exclusion criteria) are chosen to be representative of the population that the trial will be generalised to, i.e. patients undergoing gastrectomy.

3 OBJECTIVES

3.1 Primary Objective

Can enzyme supplementation with every meal after gastrectomy lead to improved disease specific quality of life at 6 months after gastrectomy?

3.2 Secondary Objectives

Can enzyme supplementation with every meal after gastrectomy lead to less weight loss and better nutritional status after gastrectomy?

4 TRIAL DESIGN AND DESCRIPTION

4.1 Trial Design

PANEM is a randomized, phase 3, parallel arm, placebo-controlled, double blinded, multi-centred, superiority trial.

Patients will be randomized 1:1 **either** to experimental (NORTASE®) **or** control (placebo) arm.

4.2 Low intervention clinical trial

This clinical trial is a low intervention clinical trial meeting the following conditions:

(a) the investigational medicinal product is authorised;

(b) according to the protocol of the clinical trial, the investigational medicinal products are used in accordance with the terms of the marketing authorisation; and

(c) the additional diagnostic or monitoring procedures do not pose more than minimal additional risk or burden to the safety of the subjects compared to normal clinical practice in any Member State concerned.

4.3 Patient Involvement

Neither patients themselves nor patient representatives from patient advocacy groups were involved in the planning and design of the clinical trial.

4.4 Trial Sites and Number of Trial Subjects

The trial is planned to be conducted in about ten trial sites in Germany.

The aim is to include a total number of 168 patients **evaluable for the primary analysis**. Assuming a drop-out rate of about 10%, a total of 188 patients are to be randomized (see 9.4).

4.5 Expected Duration of Trial

- **Duration per patient:**
  Duration of intervention: 6 months
  To obtain important survival data, **all patients** will be followed until the end of the entire trial (LPLV = last patient last visit). Survival visits will take place every 6 months until LPLV.

- **Duration of the trial:**
  First patient in to last patient out (months): 42
  Recruitment period (months): 36
  Duration of the entire trial, including preparation and analysis (months): 54

The first act of recruitment (**start of trial**) will be the date of the first release for patient recruitment.

In accordance with international guidelines, the informed consent of trial participants will be in writing (written, dated and signed by the person performing the interview referred to below, and by the subject), this will be the **start of recruitment**.

The formal end of the trial is final visit of the last patient (LPLV).

4.6 Premature Termination of the Trial

Premature termination of the trial for a single patient is described in chapter 7.6.1

4.6.1 Termination of the Trial at a Single Site

The trial can be aborted at a single site if:

- the protocol is not adhered to,
- the quality of data is deficient,
- there is inadequate recruitment.

The coordinating investigator decides whether or not to exclude the site, together with the sponsor and biometrician.

Investigators and sites no longer participating in the trial must inform the coordinating investigator immediately and should provide justification for the decision. Further treatment of patients still involved in the trial has to be arranged together with the coordinating investigator.

CAUTION: Trial site may be temporarily placed on hold or closed by the sponsor if no or insufficiently qualified personnel (investigator) is available.

4.6.2 Termination of the whole Trial or of individual Arms of the Trial

The trial can be terminated prematurely by the sponsor in the event of

- serious adverse events
- changes in the risk-benefit considerations, e.g. as a result of unexpected adverse events
- new insights from other trials
- an insufficient recruitment rate.

Regarding the safety-related aspects, the trial biometrician will monitor imbalances between the arms in SAEs that are at least possibly related to the study medication. If $p < 0.01$ according to a chi-squared test and there are at least a mean of two SAEs per patient-year and safety should favour the placebo arm, then the trial biometrician will inform the coordinating investigator to discuss termination of the trial. The final decision regarding the premature termination of the trial will be made by the sponsor and the authorised representative/coordinating investigator.

The trial must also be terminated if the approval of the responsible NCA has been revoked, and if the maximal sum insured can not be adjusted, although required.

5 TRIAL SUBJECTS

This trial includes patients who have just received a partial or total gastrectomy. The most common disease requiring gastrectomy is gastric cancer and certified centres in Germany perform an average of about 26 gastrectomies per year with this indication.[9]

The investigators in this trial have direct contact to the patients prior to the gastrectomy. Usually, potential patients will be asked about participation in the PANEM trial before gastrectomy is performed, and informed consent will be obtained. Nevertheless, eligible patients may still be included after gastrectomy, but no later than three days after beginning oral nutrition.

Given the number of patients seen at each site and the eagerness expected from a lot of patients to participate in the trial, the recruitment goals are considered realistic.

5.1 Inclusion Criteria

Definition of the trial population

1. Gastrectomy (total and partial[9])

Regulatory requirements

2. Age 18 or older
3. Written informed consent

5.2 Exclusion Criteria

Intervention impossible (due to anatomic/medical reasons)

1. Indication for pancreas enzyme therapy

Unsuitable for the trial

2. Gastrectomy with palliative intention
3. UICC (Union for International Cancer Control) Stage IV gastric malignancy unless surgery is planned according to new guidelines[10] in oligometastatic disease
4. Malnutrition of other aetiology
5. Life expectancy < 12 months

Contraindications

6. Known lactose intolerance
7. Known hereditary galactose intolerance

---

[9] At least ≥ 50 % of the stomach must be resected.

8. Patients on alpha-glucosidase inhibitors (AGIs)
9. Acute pancreatitis
10. Acute episode of chronic pancreatitis
11. Known hypersensitivity to moulds (mould allergy) or any other ingredient of NORTASE®

Regulatory requirements

12. Participation in competing interventional trials may be allowed under circumstances described in chapter see 5.4
13. Patients under legal supervision or guardianship[10]
14. Patients who are dependent on the investigator or the medical staff of the trial team or the coordinating investigator or the sponsor
15. Fertile women (within two years of their last menstruation) without appropriate contraceptive measures[11] (implanon, injections, oral contraceptives, intrauterine devices, partner with vasectomy) while participating in the trial[12] .

Others

16. Pregnant or nursing women
17. Suspected lack of compliance
18. Patients who were already enrolled in the trial

All gastrectomy patients (partial and total) are included in this trial, since the trial rationale suggests that they could all benefit from the intervention and the results are to be generalized to precisely these patients. However, patients with an indication for pancreas enzyme therapy cannot be included since placebo treatment would be unethical. Although patients with advanced disease may theoretically benefit from the intervention, they are excluded from this trial since the effects of the disease are expected to dominate well-being and mask such putative benefits. Moreover, the endpoints at 6 months are not appropriate to a palliative situation.

---

[10] incl. subjects, who are committed to an institution by virtue of an order issued either by the judicial or the administrative authorities

[11] In compliance with “Maintenance of the ICH Guideline on non-clinical safety studies for the conduct of human clinical trials for pharmaceuticals, M3(R1), CPMP/ICH/286/95, Note 3”

[12] See summary of product characteristics: “There are no concerns about use during pregnancy or breastfeeding”.

5.3 Justification for the Inclusion of vulnerable populations

This clinical trial will not include particularly vulnerable individuals as defined by EU CTR Annex I chapter D point x.

5.4 Participation in more than one Clinical Trial

During the verification of the inclusion and exclusion criteria the investigator or authorised medical staff of the trial team check if the patient is currently participating in other interventional clinical trial(s) that may either interact with the intervention of this trial or where endpoints can not be easily attributed to one or the other trial. Should this be the case, the patient will not be included.

In general, it should be noted that NORTASE® is taken orally and not absorbed into the blood stream. In particular, it does not interact with other medicinal products administered intravenously (e.g. chemotherapeutic agents).

Moreover, by signing the informed consent form, the patient confirms, if applicable, that he has informed the investigator of participation in any other competing interventional clinical trial simultaneously.

Under certain circumstances, patients may participate in the PANEM-trial; even if they are already enrolled in another trial. This applies to:

- trials in which the intervention itself is completed before the intervention in the current trial begins (e.g. clinical trials involving new surgical procedures in gastrectomy)

- clinical trials with already approved and known medicinal products (i.e. the EU-CTR definition of a “low intervention clinical trial”, e.g. known safety profile and WITHOUT possible interactions with NORTASE®).

5.5 Rationale for Sex and Age Distribution

We expect a sex ratio of about 2:3 or 3:7 (female:male).[3] Most patients are expected to be between 50 and 70 years old.[11] All patients undergoing gastrectomy that fulfil the inclusion and exclusion criteria will be informed about the clinical trial and asked to participate. We expect to be able to determine possible differences concerning the efficacy and side effects of the trial therapy depending on potential sex specific differences.

6 INVESTIGATIONAL PRODUCT

6.1 Trial Drugs

IMP

Trade Names and manufacturer:	NORTASE®, Repha GmbH Biologische Arzneimittel (Repha GmbH)
Drug allocation:	provided by Repha GmbH
Packaging:	Amber glass bottle(s) with polyethylene seal
	Units per immediate package: 100 capsules per bottle
	One bottle is in the outer package, a paper box
Storage conditions:	no special storage conditions are required
Stability:	in intact container: 3 years
	after opening: 6 months
	(see actual SmPC)

The simplified *Investigator Medicinal Product Dossier* (IMPD) contains detailed information on the investigational product. For clinical information, reference documents are the Summary of medicinal Product Characteristics (Fachinformation / SmPC).

Placebo

Manufacturer:	Repha GmbH Biologische Arzneimittel (Repha GmbH)
Drug allocation:	provided by Repha GmbH
Packaging:	Amber glass bottle(s) with polyethylene seal
	Units per immediate package: 100 capsules per bottle
	One bottle is in the outer package, a paper box
Storage conditions:	no special storage conditions are required
Stability:	in intact container: 3 years
	after opening: 6 months

The simplified *Investigator Medicinal Product Dossier* (IMPD) contains detailed information on the placebo.

All documents mentioned are part of the submission documents.

6.2 Packaging and Labelling of the Trial Drug

The investigational product and placebo will be manufactured, packaged and labelled by Repha GmbH and contracted third-party manufacturers according to the standards of Good Manufacturing Practice (GMP).

The trial drug will be labelled as required by the EU CTR 536/2014, Annex VI. The labelling will be in the official language(s) of the countries in which the trial takes place.

The master label of immediate and outer package is part of the documents that will be submitted to the responsible federal authority (see 16.3).

For each patient, the entire trial medication is packed individually in a box, i.e. this box contains a total of 28 bottles of NORTASE® or placebo.

The trial preparations will not be shipped before all requirements for the start of the clinical trial have been fulfilled according to the internal SOP. The sponsor will receive from Repha GmbH one QP release per batch and will release the batch on this basis as sponsor. The trial medication will be shipped to the trial sites upon request of the sponsor, executed by Repha GmbH. A detailed documentation of every shipment, number of boxes and capsules, which will be used in this clinical trial will be made and kept on file.

6.3 Storage and Handling Requirements

The investigator shall take responsibility for and shall take all steps to maintain appropriate records and ensure appropriate supply, storage, handling, distribution and usage of investigational product in accordance with the protocol and any applicable laws and regulations.

Clinical supplies must be stored in a secure, limited-access location under the storage conditions specified above.

Receipt and dispensing of trial medication must be recorded by an authorised person at the trial site.

Clinical supplies may not be used for any purpose other than that stated in the protocol.

6.4 Drug Accountability

Blinded trial drugs (IMP, placebo) will be provided to the trial sites by Repha GmbH. The trial drug may only be used within the framework of this clinical trial and in accordance with this trial protocol.

Initially, each trial site will receive trial medication to treat 2 patients (per patient 28 boxes à 100 capsules). Depending on the number of patients being included in the trial, each trial site will receive additional trial product after a written request to the ZKS Leipzig, which will inform Repha GmbH on the need of medication at the site.

Expired packages will be replaced.

The investigator will confirm receipt of the trial drug in writing. He/she will keep a record of the trial drug (i.e. number of boxes) dispensed to and – if applicable - returned from each patient.

This pragmatic trial does not intend to show primarily how the enzymes affect metabolism and digestion. The focus is on quality of life. Hence, an overly precise record of the intake of medication is unnecessary. Moreover, the leeway in the permitted number of capsules per meal and varying numbers of meals per day would render it very difficult to establish a target value for the number of capsules to be taken in a 3 month period. It would thus be overkill to request from patient, site and monitor that a precise record of hundreds of unused capsules be maintained. The measures of compliance to be used in this trial are described in 6.5.2.

Upon completion or termination of the trial, all unused and returned investigational product will be destroyed at the site per institutional policy provided that procedures for proper disposal have been established according to applicable federal, state, local and institutional guidelines.

6.5 Administration of Trial Drug

6.5.1 Procedures

According to SmPC, patients should take 5-15 capsules of NORTASE® per day, distributed with meals (1-3 capsules per meal). Gastrectomized patients also typically eat five or more small meals per day instead of three.

Patients start in the hospital on **day 0** with an initial dose of 5 (meals) x 2 capsules taken with meals; i.e. 10 capsules per day. At the three-weeks-visit 2, if necessary, the dosage can be increased to 5 (meals) x 3 capsules; i.e. 15 capsules per day, if no sufficient effect can be observed. This correlates with the approved maximum dose of NORTASE®. The entire amount of capsules (in 28 boxes) should be taken by the patient. Ideally, the amount should be sufficient until visit 4.

It is possible to reduce the dosage without the patient stopping the treatment.

The hard capsules are ingested whole with sufficient liquid (water, juice or warm tea) during a meal or snack.

NORTASE® contains active enzymes which, when released in the oral cavity, e.g. by chewing, may cause ulceration of the oral mucosa. Care should therefore be taken to swallow NORTASE® whole.

6.5.2 Compliance

At day 0 and at visit 3, the patients receive the amount of the trial medication required until the next visit (including a reserve). The patient notes the daily intake (i.e. number of capsules) in the subject diary to be provided by the sponsor. The investigator examines the record at each visit and takes back empty, half-full packages or unused medication.

The number of daily taken capsules is documented in the CRF. Based on this, an assessment regarding intake will be made in following categories:

Always: > 95%

Often: approx. 75%

Sometimes: approx. 50%

Seldom: approx. 25%

Never: < 5% of the expected amount of capsules taken.

6.5.3 Dealing with Side-effects

Known side effects are the following according to SmPC: In rare cases, nonspecific concomitant symptoms such as diarrhea, nausea, obstipation, and upper abdominal

discomfort, as well as allergic respiratory and skin reactions have been observed following occupational sensitization with mould enzymes.

Emergency measures need not to be defined. The patient may reduce the dosage after consultation with the trial physician without the patient stopping the treatment.

6.5.4 Alternative Medication

Only the trial medication may be administered.

6.5.5 Contraindicated/Forbidden Concomitant Medication

No contraindicated / prohibited concomitant medications are described in the SmPC of NORTASE ®.

6.5.6 Overdose and Abuse

So far, no cases of overdose and abuse have been reported.

6.6 Blinding and Unblinding

The trial is a double-blind trial. The trial medication and placebo will be manufactured by Repha GmbH. Blinding will also be conducted by Repha GmbH following a randomisation list provided by ZKS Leipzig.

In order to preserve the blinding of the trial, a minimum number of staff will see the randomisation table and treatment assignments before the trial is complete.

Information regarding the treatment arm of a patient will be kept in a sealed and light-proof envelope (emergency envelope). The envelopes will be kept at ZKS Leipzig, and each trial centre will receive the emergency envelopes for the patients treated at the site as soon as the medication is shipped to the trial site by Repha GmbH. At the trial site, the envelopes have to be kept in the investigator site file (ISF). The hand-out of emergency envelopes as well as the return of unopened envelopes will be documented on special form sheets.

Unblinding for adverse events may be performed ONLY if an inevitable, intermediate medical intervention due to safety reasons requires knowledge of the patient’s treatment assignment. In case of emergency, immediate unblinding procedures will be initiated by the investigator at the trial site without the need to contact other persons. If, however, in the assessment of the investigator, immediate unblinding is not necessary, then it would be advisable to discuss the assessment regarding unblinding with the coordinating investigator.

If an investigator, site personnel performing assessments, or patient is unblinded, the patient remains in the trial unless exclusion is deemed necessary for safety reasons by the coordinating investigator.

If an investigator opens an emergency envelope:

- he has to document date and time of unblinding on the emergency envelope and sign it
- the opened envelope has to be kept in the ISF
- unblinding has to be documented in the patient’s file as well as on the (e)CRF

Unblinding in case of suspected unexpected serious adverse reactions (SUSARs) will follow the SOPs of the ZKS Leipzig and will be described in separate working instructions of the pharmacovigilance department. In case of unblinding by the pharmacovigilance department of the ZKS Leipzig, this will be documented in the pharmacovigilance database.

7 INDIVIDUAL TRIAL PROCEDURES

7.1 Patient Information and Informed Consent

The investigators or authorised medical staff of the trial team will seek the patient's consent before performing any trial specific medical procedures with the patient. Patients who are considered not competent to consent to participate, e.g. due to an insufficient level of understanding, cannot participate in the trial.

In accordance with international guidelines, the informed consent of trial participants will be in writing (written, dated and signed by the person performing the interview referred to below, and by the subject).

The patient's consent must refer explicitly to the collection and processing of health-related data. Therefore the patient should be informed explicitly about the purpose of collecting the data and scope of what is to be collected and that personal data, including health related data, will be stored and used for analyses in a pseudonymized form.

Before obtaining informed consent, the potential trial participant will receive information regarding the clinical trial in an interview. It will be performed by a qualified medical member of the trial group authorised by the investigator for this task.

The interview has to cover the following items:

- the nature, objectives, benefits, implications, risks and potential inconveniences of the clinical trial
- the expected duration of the subject's participation in the clinical trial
- the information that the patient may withdraw his/her consent to participate at any time without giving reasons. The patient is to be informed that in case of revocation of his/her consent, the stored data may be used further, as may be necessary to
    - assess effects of the medicinal product being tested,
    - guarantee that the patient's personal interests are not adversely affected,
    - comply with the requirement to provide complete authorisation documentation.
- potential treatment alternatives
- follow-up measures in case of early termination of the trial for the patient or overall
- the applicable damage compensation system in case of damage to a patient
- the right on data access, rectification and withdrawal of personal data

The subject will have the opportunity to ask questions at any moment.

Adequate time will be provided for the subject to consider his or her decision.

7.1.1 Withdrawal of Informed Consent

Patients may withdraw their consent to participate at any time without giving reasons. Nevertheless, the patient should be asked for the reason of the premature termination after being informed that he does not need to do so. Information as to when and why a patient was registered/ randomised and when he withdrew consent must be retained in the documentation.

The patient is to be informed that in case of revocation of his/her consent, the stored data may be used further (according to § 40 lit 2a sublit 3 German Medicinal Products Act), as may be necessary to

- assess effects of the drug being tested,
- guarantee that the patient's personal interests are not affected adversely,
- comply with the requirement to provide complete authorisation documentation.

Data no longer required for the aforementioned purposes shall be deleted immediately.

7.2 Enrolment in the Trial

Patients will exclusively be recruited at the participating trial sites, which are all specialised in gastrointestinal disorders, nutritional medicine and care of patients after operations on the gastrointestinal tract.

Potential patients will be informed about the PANEM trial during their inpatient stay **before or after gastrectomy**. Procedures performed solely for the clinical trial can be performed only after obtaining informed consent.

Generally, subjects are enrolled in the trial by the local investigator or authorised medical staff at the trial sites. After informed consent is given by the patient, general inclusion/exclusion criteria are checked as well as the demographic data.

NOTE: Registration must take place before randomisation and start of any trial therapy.

The registration is performed via a web-based tool that is provided by the Clinical Trial Centre Leipzig. The registration is forwarded back automatically to the investigator and the Clinical Trial Centre Leipzig (email).

7.3 Screening (max. 30 days before gastrectomy until visit 1)

In order to check all inclusion and exclusion criteria, several examinations including blood sampling/laboratory analyses during the patient's screening investigation - **before randomisation** - are necessary.

Screening visit (Scr) takes place with a maximum of 30 days before gastrectomy until Day 0 (= visit 1; max. 3 days after begin of oral nutrition).

Screening / Baseline assessments

- Assessment of medical and medication history
- Assessment of demographic data
- Clinical assessment
    - Assessment of vital signs (arterial blood pressure, heart rate, temperature, body weight)
- Local lab analysis
    - Including serum haemoglobin, total protein, albumin, glucose, HbA1c, cholesterol, vitamins A, B12, D, E and K

    - Note!

      Glucose, HbA1c, cholesterol, vitamins A, B12, D, E and K only need to be measured once, either for visit Scr or V1 (see 7.5)

    - pregnancy test – in blood or urine

- Quality of life Questionnaires
- Further **optional** diagnostic procedures; for all patients at selected trial sites
    - Faecal elastase-1 test

As soon as all assessments required for the final check of eligibility are available**,** i.e. in all cases **after** gastrectomy, the patient will be randomized and the baseline assessments will be completed and recorded in the eCRF.

If the patient meets all inclusion criteria and none of the exclusion criteria, the randomisation will be performed.

The randomisation result will be shown online by the randomisation tool and an automatic e-mail will be sent to the trial site for filing in the investigator site file (ISF).

In cases, when the internet-based tool is not available (e.g. server shut down, internet connection blocked), the randomisation page of the eCRF can also be filled in by hand and sent to the ZKS Leipzig by e-mail (scan) or fax. There will be a randomisation list at the ZKS Leipzig allowing database independent randomisation during regular office hours (Monday – Friday, between 8:00 am and 5:00 pm). After such a randomisation, the patient will be entered to the database by the ZKS Leipzig as soon as the database is functional again.

Randomisation results and allocated treatment has to be documented in the patient's medical record.

7.3.1 Discovery of a Violation of the Eligibility Criteria after the Fact

In general, the violation of eligibility criteria is not a reason for premature withdrawal of the patient from the trial therapy or from the whole trial.

If a violation of a selection criterion is discovered after registration/randomisation of a patient, this has to be documented in the eCRF, which will result in an automatic report to the responsible trial team members at the ZKS Leipzig.

After consultation with the coordinating investigator and biometry, the project management of the ZKS Leipzig informs the investigators or authorised medical staff immediately of the decision regarding the further treatment of the patient. Treatment should always be continued unless a substantial safety risk is to be feared. The patient's data will continue to be recorded unless the patient revokes his/her informed consent. For procedures after premature trial termination of a single patient see chapter 7.6.1.

7.4 Description of the Treatment Procedures

7.4.1 Intake of NORTASE® versus placebo

After randomisation at the latest on day 0 the patient will be treated in accordance with the

allocated treatment arm: **intake of NORTASE®** (experimental arm) or **intake of placebo** (control arm).

The **first intake** of trial medication will be administered at **day 0** in the hospital, i.e. **not later than three days** after the re-start of oral nutrition (details see 6.5.1).

7.4.2 Standard treatment

Standard treatment has to be started/continued in both treatment groups, and should follow national guidelines, if not available international guidelines.

7.5 Description of Trial Visits

In both trial arms, regular visits are scheduled as follows:

Treatment phase

- Visit 1 [day 0]
- Visit 2 [week 3 +/- 1 week]
- Visit 3 [month 3 +/- 2 weeks]
- Visit 4 [month 6 +/- 4 weeks]

<u>At visit V1</u> the following procedures will be performed:

- Assessment of concomitant medication unrelated to the gastrectomy
- <u>Clinical assessment</u>
    - Assessment of vital signs (arterial blood pressure, heart rate, temperature, body weight)
- <u>Local lab analysis</u>
    - Including serum haemoglobin, total protein, albumin, glucose, HbA1c, cholesterol, vitamins A, B12, D, E and K

In cases where screening visit does not occur until **day 0**, visit V1 is **not required**.

<u>At visit V2</u> the following procedures will be performed:

- Assessment of adverse events and concomitant medication unrelated to the gastrectomy
- Quality of life Questionnaires
- Assessment of survival status
- Basic questions on eating habits and nutrition (including the use of supplementation)

In some cases, visit 2 can only be carried out by telephone because the patient is in rehabilitation, for example.

<u>At visit V3</u> the following procedures will be performed:

- Assessment of adverse events and concomitant medication unrelated to the gastrectomy
- Basic questions on eating habits and nutrition (including the use of supplementation)
- Distribution of trial medication and drug accountability
- Assessment of survival status

- <u>Clinical assessment</u>
    - Assessment of vital signs (arterial blood pressure, heart rate, temperature, body weight)
- <u>Local lab analysis</u>
    - Including serum haemoglobin, total protein, albumin, glucose, HbA1c, cholesterol, vitamins A, B12, D, E and K
- <u>Quality of life Questionnaires</u>

<u>At visit V4</u> the following procedures will be performed:

- Assessment of adverse events and concomitant medication unrelated to the gastrectomy
- Basic questions on eating habits and nutrition (including the use of supplementation)
- Drug accountability
- Assessment of survival status
- <u>Clinical assessment</u>
    - Assessment of vital signs (arterial blood pressure, heart rate, temperature, body weight)
- <u>Local lab analysis</u>
    - Including serum haemoglobin, total protein, albumin, glucose, HbA1c, cholesterol, vitamins A, B12, D, E and K
- <u>Quality of life Questionnaires</u>
- <u>Further **optional** diagnostic procedures; for all patients at selected trial sites</u>
    - Faecal elastase-1 test

<u>Survival visits (SV):</u>

After Visit 4 telephone visits will take place every six months (+/- 4 weeks) until the end of the overall trial (last patient last visit) to assess the survival status of patients.

7.6 Premature Termination of the Therapy or Follow-up

The primary statistical analysis follows the intention to treat principle as closely as possible. For a valid analysis, it is of great importance to minimise the rate of drop-outs. Therefore, in patients who do not withdraw their consent, all trial visits shall be performed as scheduled.

7.6.1 Premature Termination of the Therapy for Individual Patients

Trial therapy (i.e. treatment with IMP resp. placebo) must be terminated prematurely, if the investigator decides/judges that

- an adverse event occurred to a patient and continuation of trial treatment would thus be an unacceptable risk for this particular patient
- the worsening of a patient (who is not responding to treatment) contraindicates the treatment with trial therapy transiently or permanently
- any other reason of medical prudence applies

Trial therapy (i.e. treatment with IMP resp. placebo) must also be terminated prematurely

- on request of the patient

With exception of the rules described above, premature termination of therapy should be avoided. In case the patient misses the scheduled visits, the investigator may contact the patient directly, in order to motivate him/her to continue.

In case of premature termination of therapy, it is necessary to document the date (as exactly as possible), the reason of termination and the current condition of the patient.

All further trial visits until month 6 will take place as planned and described in section 7. **Termination of trial therapy does not mean that the patient is off-trial.**

7.6.2 Premature trial termination for individual patients

All randomised patients will be followed up until month 6, and thereafter to the end of the overall trial (LPLV) for survival status.

Premature termination of trial therapy does not necessarily lead to individual trial termination (see chapter 7.6.1 for explanation).

The only circumstances in which a premature trial termination (i.e. no further trial visits) in a randomised patient is unavoidable are:

- a patient is worsening (and not responding to treatment) that contraindicates permanently participation in any trial visits
- withdrawal of informed consent,
- complete loss of contact to the patient or
- death of the patient.

In case of premature termination of trial, it is necessary to document the date (as exactly as possible), the reason of termination and the current condition of the patient. Therefore, the eCRF “end of study (ES)” has to be completed for each patient. Data entry to this eCRF page will trigger an automatic report to the responsible trial team members at the ZKS Leipzig.

8 ADVERSE EVENTS (AE/SAE)

8.1 Adverse Events (AE)

8.1.1 Definition

According to EU CTR Art. 2 (2) point 32 'Adverse event' means any untoward medical occurrence in a subject to whom a medicinal product is administered and which does not necessarily have a causal relationship with this treatment.

Adverse Events encompass any unfavourable and unintended sign (including an abnormal laboratory finding, for example), symptom, or disease that arise newly or worsen after the inclusion of the patient into the trial.

8.1.2 Critical Adverse Events

Not applicable

8.1.3 Documentation and Reporting

Adverse events will be documented on special AE-forms from **start of the therapy (day 0)** until final visit (V4) according to the detailed guidance on the collection, verification and presentation of adverse event/reaction reports arising from clinical trials on medicinal products for human use.

At each visit, the patient will be asked a non-leading question such as "Have you had any health problems since the last visit". All AEs reported in response to questioning, as well as AEs reported spontaneously will be recorded on the 'adverse event' page(s) in the CRF, regardless of causality. In addition, the patient will be trained to inform the clinical trial site of any health problems arising between visits by phone or personal visit.

If an AE fulfils any of the criteria for an SAE (see chapter 8.4.1 for SAE-definition), both the AE pages of the CRF **and** the SAE form must be completed. This applies to all SAEs, **whether or not they are considered to be related to trial treatment.**

For both serious and non-serious AEs, documentation should be supported by an entry in the patient's heath record. Required information in the patient's health record should include:

- type of AE
- grade/severity/intensity acc. to e.g. NCI CTCAE V5.0
- seriousness (see also section 8.4)
- onset date
- end date
- actions required
- outcome
- assessment of its relationship to trial drug

All abnormal physical and/or laboratory results which are considered to be clinically relevant by the investigator should be recorded as AEs. If an abnormal laboratory result meets any of the criteria for a SAE, this must also be reported on the SAE Form.

Adverse Events are classified by their seriousness, intensity/severity and relationship to the IMP (see also 16.1)

The following are **exceptions from the documentation of (serious) adverse events** as they represent typical complications of the gastrectomy and following treatments (e.g. chemotherapy and/or radiation)**:**

- bleeding as a result of gastrectomy,
- wound healing disorders as a result of gastrectomy,
- infections as a result of gastrectomy,
- events resulting of re-gastrectomy incl. post-treatment
- events resulting of radiation / chemotherapy as a result of the underlying disease

All other Adverse Events should be documented on the specific (S)AE forms, especially, but not only, if the investigator deems the event as related to the study drug.

8.2 Safety Analysis

During the course of the trial, every patient will be monitored closely. On every visit this encompasses documenting Adverse Events

8.3 Concomitant Diseases

Concomitant diseases will be recorded at baseline. Any worsening of concomitant diseases will be regarded as adverse event.

8.4 Serious Adverse Events/Serious Adverse Reactions (SAE/SAR)

8.4.1 Definition

According to EU CTR Art. 2 (2) point (33) ‘Serious adverse event’ means any untoward medical occurrence that at any dose requires inpatient hospitalisation or prolongation of existing hospitalisation, results in persistent or significant disability or incapacity, results in a congenital anomaly or birth defect, is life-threatening, or results in death;

Medical and scientific judgement should be exercised in deciding whether expedited reporting is appropriate in other situations, such as important medical events that may not be immediately life-threatening or result in death or hospitalisation but may jeopardise the patient or may require intervention to prevent one of the other outcomes listed in the definition above. These should also usually be considered serious.

Exceptions from SAE documentation and reporting are defined in chapter 8.1.3.

8.4.2 Documentation and Reporting Obligations: INVESTIGATOR

Serious Adverse Events have to be documented on the SAE-forms from start of therapy until final visit (V4) and the investigator must report them to the sponsor immediately. If more information about the SAE becomes available later, it must also be reported to the sponsor immediately.

In the event of a patient's death, the investigator/deputy or the authorised medical staff provides ethics committee, the responsible federal authority and the sponsor with all further information needed to fulfil their tasks **upon request**.

In all the reports, personal data are to be pseudonymized by using the patient's identification code. It must be possible to relate the initial and all follow-up reports to each other by means of the patient identification number, name and address or the like.

The investigators or the authorised medical staff must report every Serious Adverse Event as soon as getting knowledge of it to the following address:

ZKS Leipzig/Pharmacovigilance
Universität Leipzig
Zentrum für Klinische Studien Leipzig
Haertelstr. 16-18, 04107 Leipzig
Phone: +49/341/97-16129
E-mail: pharmacovigilance@zks.uni-leipzig.de

Fax: +49/341/97-16278

8.4.3 Serious Adverse Events without immediate reporting

Not applicable

8.4.4 Documentation and Reporting Obligations: SPONSOR

After the ZKS Leipzig receives the SAE, it is immediately passed on to the coordinating investigator/responsible person for medical assessment.

The coordinating investigator/responsible person forms a second medical opinion of the SAE with respect to causal relationships and the decision as to whether or not it was expected, as described in chapter 16.1.3 and 16.1.4 and forwards the second opinion to the ZKS Leipzig within two days of its arrival.

In the ZKS Leipzig the SAE data are entered into the SAE database immediately and the MedDRA coding takes place simultaneously.

Then forwarding as per law and as described in Chapter 8.6 only for Suspected Unexpected Serious Adverse Drug Reactions (SUSARs) takes place.

Details of the sponsor's documentation and reporting obligations will be specified in a special, trial-specific pharmacovigilance plan, which will be written and finalised alongside with this protocol, if possible.

8.5 Periodic Reports

8.5.1 Annual Safety Report

According to EU CTR the sponsor creates a safety report annually (Annual Safety Report,

ASR[13]) and sends this report via CTIS to the European Medicines Agency.

The obligation to prepare an ASR starts with the first authorisation of the clinical trial in accordance with this Regulation. It ends with the end of the last clinical trial conducted by the sponsor with the investigational medicinal product.

The ASR will be prepared by the ZKS Leipzig (project manager, responsible biometrician and PV data manager) acc. to the national requirements. Deadlines regarding the ASR are based on the EU CTR.

The final report will be released in co-operation with the coordinating investigator/sponsor's representative.

8.5.2 ASR for all IMPs in the clinical trial

The sponsor will provide a single safety report on all investigational medicinal products used in the clinical trial in accordance with Article 43 (2) EU CTR, since the sponsor is not the marketing authorisation holder for the investigational medicinal products.

8.6 Suspected Unexpected Serious Adverse Reactions (SUSAR)

8.6.1 Definition

Suspected Unexpected Serious Adverse Drug Reactions (SUSARs) are side-effects (probably or definitely connected with the administration of the investigational product), the nature or severity of which are inconsistent with the information available about the product. Information about the trial product is contained in the Investigator's Brochure or the SmPC (Summary of Product Characteristics).

8.6.2 Documentation und Reporting Obligations

Information for SPONSOR

The sponsor submits all information available about a SUSAR immediately to the European Medicines Agency, at the latest within 15 calendar days after the event becomes known – according to EU-CTR.

For every SUSAR that results in death or a life-threatening condition, the European Medicines Agency must be informed by the sponsor within 7 calendar days after the event becomes known. Additional information has to be given within 8 further calendar days

Details of the sponsor's documentation and reporting obligations will be specified in a special, trial-specific pharmacovigilance plan, which will be written and finalised alongside with this protocol.

Before the SUSAR reporting an unblinding must be performed.

[13] See "Detailed guidance on the collection, verification and presentation of adverse reaction reports arising from clinical trials on medicinal products for human use").

Information for INVESTIGATOR

The investigators will be informed about the SUSARs not as individual case safety reports but on a regularly base (SUSAR-listing). In case of findings that could adversely affect the safety of the subjects a single case report will be submitted to the investigators. This has to be confirmed by the investigator in writing.

Further details will be specified in the trial-specific pharmacovigilance plan.

To ensure that the investigators and all other trial personell remain “blind” in the case of a SUSAR, the investigating centres will receive all potential SUSARs without information concerning the administered drug (i.e regardless if the patient was treated with verum or placebo).

8.7 Follow up of Adverse Reactions

Details of the sponsor’s trial specific follow-up strategy will be specified in a special, trial-specific pharmacovigilance plan, which will be written and finalised alongside with this protocol

8.8 Other Safety Relevant Issues

There may be other events which are relevant in terms of benefit-risk balance and which should be reported in a timely manner to the Member States concerned. It is important for subject safety that, in addition to serious adverse events and reactions, all unexpected events that might materially influence the benefit-risk assessment of the medicinal product or that would lead to changes in the administration of a medicinal product or in overall conduct of a clinical trial are notified to the Member States concerned.

Examples of such unexpected events include

- an increase in the rate of occurrence of expected serious adverse reactions which may be clinically important,
- a significant hazard to the patient population, such as lack of efficacy of a medicinal product,
- or a major safety finding from a newly completed animal study (such as carcinogenicity).

The sponsor decides if such an event like listed above comprise a safety issue and must be reported.

8.9 Therapeutic Procedures.

If a patient requires treatment as a result of an Adverse Event, then it must meet the recognized standards of medical care in order to restore the patient’s health. Appropriate resuscitation devices and medication must be available in order to treat the patient as quickly as possible in the event of an emergency.

The action taken to treat the AE/SAE must be documented by the investigator either in the appropriate CRF and/or using additional documents.

8.10 Dealing with Pregnancy

Female trial participants of childbearing potential must immediately inform the trial site if they

become pregnant during the course of the trial. The investigator will exclude the trial participant immediately from the trial. Every pregnancy that occurs while taking part in the trial must be reported to the ZKS Leipzig by the investigator/deputy or the authorised medical staff within 24 hours of having learned of it by informal communication:

ZKS Leipzig / Pharmacovigilance
Universität Leipzig
Zentrum für Klinische Studien Leipzig
Härtelstr. 16-18, 04107 Leipzig
Telefon: +49/341/97-16129
Fax: +49/341/97-16278
E-mail: pharmacovigilance@zks.uni-leipzig.de

The ZKS Leipzig will then supply the essential forms for documentation to the investigator/deputy investigator without unduly delay. Pregnancies that occur in female trial subjects have to be reported by using the form: “Report on the arising of a pregnancy during exposition to a trial medication”. Severe side effects and complications during a pregnancy as well as congenital birth defects are Serious Adverse Events by definition and therefore also have to be reported on the Serious Adverse Event form according to the reporting procedures described above.

The outcome of a pregnancy has to be reported on the form “Report on the outcome of a pregnancy during/after exposition to a trial medication”. This form will document the outcome of the pregnancy, including a spontaneous or voluntary abortion, details of the birth process, the presence or absence of congenital malformations and birth defects, maternal or foetal complications and the potential relationship to the trial drug.

Collecting data concerning the outcome of a pregnancy is only permitted if the trial subject puts down her permission in writing beforehand. The collection of data on the child's health requires the prior information and consent of the guardian/parents (usually both).

9 BIOMETRY

9.1 Biometrical Aspects of the Trial Design

This is a multi-centre, parallel group, double-blinded randomized, controlled superiority trial.

Blinding was chosen since eating behaviour and quality of life may be effected by the belief that one is or is not receiving treatment.

Recruitment will proceed until the pre-determined sample size is reached. If recruitment is considerably slower than anticipated, than a pooled estimate of variance and of drop-out/missing data can be used to see if a power of 80% can be attained with a smaller sample size. If so, then recruitment can be terminated after reaching the adjusted sample size, which will be considered a substantial amendment.

9.1.1 Measures to Prevent Bias

Randomisation

Patients will be randomized in a 1:1 ratio after gastrectomy and before beginning with oral intake of food. We randomize after surgery to exclude allocating patients with intra-operative mortality and severe complications. Patients will be randomized using variable length block randomization, stratified by trial site. It is not logistically feasible in this trial to account for other stratification variables, but relevant risk variables will be taken into account in the analysis methods.

Blinding

Knowledge of treatment can have large effects on endpoints such as quality of life, making it necessary to blind patients. Although weight and laboratory measurements are not affected by this knowledge, blinding physicians provides the simplest way to ensure that information about the randomisation result is not passed on inadvertently to the patient. Hence, we have opted for a double-blind trial design.

9.2 End Points

9.2.1 Primary End Point

Disease specific quality of life (QOL) at 6 months according to the physical sub-score of the EORTC-QLQ-C30.

Quality of life is accepted as an important marker of therapeutic options in oncology and the EORTC-QLQ-C30 is an established international instrument,[12] which has been validated in a German population.[13] The physical sub-score is chosen for two reasons. Firstly, there is evidence that this is one of the aspects of QoL most affected by gastrectomy.[14] Secondly, this is the sub-scale based on the highest number of questions, thus rendering it the most stable of the sub-scales.

9.2.2 Secondary End Points

Supportive information regarding the primary endpoint

- Sub-scales of QOL at three and six months (GILQI, EORTC-QLQ-C30 / STO22): these are accepted and validated QOL instruments in the patient population for this trial[14–19]

These endpoints provide a deeper understanding of changes in quality of life over time and focus on different components of it.

Additional endpoints relating to nutrition and weight loss

- proportion of patients who receive supplemental nutrition after the run-in period of 3 weeks or have > 10% weight loss at six months. The 10% mark is associated with clinical outcomes and is commonly used in this context, see e.g. [20] Supplemental nutrition is defined as regular use of high-caloric drinks or vitamins if advised to do so by a physician or dietary expert.
- % weight change at six months: this important marker of health status is commonly used in research in the patient population[15,19,21–24]

Endpoints related to metabolism and malnutrition

- blood parameters at six months (serum haemoglobin, total protein, albumin, glucose, HbA1c, cholesterol, vitamins A, B12, D, E and K): meta-analyses show that these parameters are used in research on nutritional status in this patient population.[16,23] Fat malassimilation can cause malabsorption of fat-soluble vitamins
- **Optional**, all patients at selected trial sites : faecal elastase-1 test (FE-1) at six months: this parameter is used in research to quantify pancreatic enzyme effects[25,26]

Descriptive endpoint

- mortality: although no effect from the intervention is expected, this is clearly an outcome of great interest. Patients will be contacted at regular intervals to acquire mortality data including date of death until last patient-out.

All secondary endpoints are exploratory and no adjustment for multiplicity is foreseen. If the primary endpoint is not significant, they do not suffice to demonstrate the effectiveness of the therapy, but may provide evidence suggesting it.

9.3 Statistical Description of the trial hypothesis

$$H_0: \mu_I = \mu_c$$

$$H_A: \mu_I \neq \mu_c$$

μ_I refers to the mean value of the physical sub-score of the EORTC-QLQ-C30 at 6 months and μ_c to its mean value in the control group. A two-sided test will be used with a significance level of 5%.

9.4 Sample Size Discussion

Estimate of Effect Size

The minimal clinically important difference (MCID) for QOL sub-scales from the EORTC-QLQ-C30 is often taken to be half of the standard deviation (SD) or 10 points across a range of cancers.[27–29] Table 2 from Avery et al. reports on patients similar to those in this trial and suggests that QOL for the physical function may be around 80-85 points at baseline with an SD of about 23.[14]

Statistical Errors

For the primary endpoint, we strive for power of 80% at a significance level of 5%.

Drop-outs

The anticipated drop-out rate is low, e.g. after one year, there were no drop-outs amongst 43 randomized gastrectomy patients in Catarci et al.[7] Moreover, mortality can be expected to be under 5% at 6 months in patients with curative gastrectomy.[30–32] However, we allow for 10% missing values in the questionnaire used for the primary endpoint. Patients who terminate use of the medication/placebo, will be retained in the final primary ITT analysis. Patients who die before reaching the primary endpoint will be replaced by newly randomized patients and if data for the primary endpoint are missing from more than 10% of the patients, additional patients may be recruited.

Sample Size

To detect an MCID of 10 with an SD of 23 using a two-sided t-test with 80% power at the 5% significance level, 84 patients per group need to be analysed (calculated with prop.t.test in R). After accounting for drop-outs, we plan to recruit 188 patients in total.

Further considerations of power

For the primary endpoint, the adjusted analysis used in the final analysis is expected to have somewhat higher power than the t-test used for the sample size determination.

Weight-loss is a common endpoint of interest in the context of gastrectomy and we therefore consider what effects are typical in a control group and what power is available to detect deviations from it. We reiterate that as an exploratory endpoint, we neither expect high power for weight-loss nor can a confidence interval excluding zero be considered confirmatory. Regarding the expected size of effects in the control arm, meta-analytical techniques based on the trials identified in Lee *et al*,[16] show that at 6 months, patients with partial gastrectomy can expect weight loss of 12.2% (95% CI 10.2% to 14.1%) and with total gastrectomy 14.5% (95% CI 12.0% to 17.0%), implying a pooled weighted mean weight loss of 14.0% with SD of 22.5%. With the sample size proposed after drop-out, power is 50% if the true difference in weight change between groups is 6.8 percentage points and would have to be 9.8 percentage points to have 80% power. Assuming that about 16% of patients lose > 10% weight at 6 months based on Fukuda *et al,*[4] then power of 50% is achieved with the proposed sample size if only 6.5% of the intervention group can be expected to reach this endpoint and would have to be only 3.3% to reach 80% power. Because of these low expected powers, the endpoints for weight loss will be estimated along with 95% CI, but no formal statistical test will be provided.

9.5 Statistical Methods

9.5.1 Analysis Population

The primary analysis will be based on the “modified Full Analysis Set” (mFAS), consisting of all randomized patients alive at 6 months and using the randomisation arm they were allocated to, irrespective of compliance. As noted above, patients will only be recruited for this trial if the gastrectomy is curative and their prognosis is fairly good, meaning that mortality within 6 months will be very rare. If a patient should die before reaching the primary endpoint, this patient will be excluded from the analysis since the gastric cancer is expected to dominate quality of life as opposed to the gastrectomy, meaning the therapy is not expected to be particularly effective, the data are uninformative and the imputation techniques used otherwise may well induce bias since such patients are clearly Missing Not At Random (MNAR).

Self-reported medication use, attending study visits and providing valid QoL questionnaires will by used as measures of compliance. Patients will be retained in the “Per Protocol Set” (PPS) if they used their medication “often” or “always” at each of V2, V3 and V4 and provided valid QoL at these visits. If there should be an administrative mix-up and a patient was given placebo instead of verum or *vice versa* then such patients will also be excluded from the PPS. The primary endpoint will be analysed for the PPS if the number of patients in at least one arm should deviate by more than 10% from the mFAS. Otherwise, a PPS analysis of the primary endpoint is optional.

Safety analyses will be conducted for all patients that received at least one dose of placebo/medication. Patients will be analysed according to the treatment received.

9.5.2 Planned Methods for Analysis

A linear mixed repeated measures model will be used to analyse the primary endpoint. The dependent variable is the physical sub-score of the EORTC-QLQ-C30 at 3 and 6 months and its value at baseline as well as (a) sex, (b) partial vs total gastrectomy, (c) previous/planned chemotherapy vs none and (d) pre-operative BMI ≥ 25 kg/m² and (e) the time point 3 or 6 months as a categorical variable. These variables were chosen based on risk factors for post-gastrectomy weight loss.[33] Trial site will be treated as a random effect. Missing data are accounted for implicitly by the mixed model. The contrast at 6 months between the arms constitutes the primary endpoint and the estimate will be accompanied by a 95% Wald confidence interval and the p-value dual to it. A linear model with complete data and without consideration of the trial site will serve as a sensitivity analysis. A second sensitivity analysis will repeat the primary analysis after including patients who have died and setting their missing data to the overall lowest observed value for the endpoint (i.e., the lowest value among all patients). Descriptive statistics will be used for the distribution of the cumulative dose of medication, separated by arm. The association with the primary endpoint will also be provided descriptively. The primary analysis does not include dose, since the primary goal of the trial is to demonstrate effectiveness and not efficacy (dose modifications are routine and thus belong to an assessment of effectiveness). Moreover, a dependence on dosage would be extremely difficult to interpret, since higher doses may be the causal result of patient dissatisfaction/discomfort, which would in turn be reflected in the primary endpoint.

Secondary endpoints for continuous data will be analysed along the same lines as the primary analysis, but without the corresponding sensitivity analysis. For binary variables (e.g. 10%

weight loss at six months) an analogous generalized linear mixed model with a logit link function will be used and a profiling method to construct confidence intervals. The p-values will be complementary to the confidence intervals. Mortality for the FAS (including patients who die before six months) will be presented graphically with a Kaplan-Meier curve. Since the intervention is not intended or expected to affect mortality and since the trial is underpowered for this endpoint, no formal statistical test of mortality is foreseen. However, a Cox regression using the same covariates as with the primary endpoint will be performed to estimate the hazard ratio and provde a 95% confidence interval for it.

The Statistical Analysis Plan will be finalized in this double-blind study before treatment assignments are revealed.

9.6 Statistical Monitoring

The trial conduct will be closely supervised by means of central and statistical monitoring according to ICH E6(R2). The objectives are

- to detect safety relevant signals as soon as possible
- to detect non-compliance and relevant protocol violations and to prevent their future occurrence by prompt reaction
- to prevent missing visits or measurements by prompt reminders
- to ensure that medication logistics is proceeding as planned
- to ascertain if replacement patients are required

Therefore, the following issues will be monitored:

- With regard to safety
  - o Patients with treatment discontinuation due to adverse events
  - o Serious adverse events
- With regard to protocol compliance
  - o Patients with major or minor findings concerning the informed consent documents as detected by on site monitoring
  - o Patients who claim to take medication less than “often” or who do not provide this information
- With regard to missing information
  - o Missing visits or visits outside schedule
  - o Missing or invalid QoL questionnaires
  - o Missing or implausible weight
- With regard to medication logistics
  - o Comparison of the allocated and allotted medication numbers
- With regard to patient replacement

- o Tally of deaths, should they occur
- o The number of missing data for the primary endpoint

This is not an exclusive list. Statistical monitoring will be continuously adapted in response to new problems/risks which might arise during trial implementation.

Detailed information about the statistical and central monitoring incl. processes, content, endpoints, data to be observed, time lines, reports and descriptive statistics will be given in a central monitoring plan.

9.7 Interim Analysis

No interim analysis is planned.

9.8 Final Analysis

The final analysis will be performed after last-patient-out, once the queries in the data base have been resolved and the SAP has been finalized.

10 DOCUMENTATION

10.1 General information and Access Rights

In the context of a database for electronic data capture only, the Case Report Form (CRF) will be designed by the ZKS Leipzig in cooperation with the sponsor’s authorised representative and provided as electronic form (eCRF). In order to facilitate the documentation as per protocol in case of malfunction of the electronic system or any of its components, a paper version of the CRF (interim CRF) will be provided in the ISF (investigator site file). The data on this paper version will be transferred to the eCRF as soon as the electronic system is available again.

Special CRF forms will be provided as paper CRF:

- **SAE-forms**, because these have to be sent to the ZKS Leipzig in a printable version for submission to the relevant authorities
- **Patient questionnaires** (e.g. for quality of life) since these will be filled in by the patient directly / will be completed by authorized staff members together with the patient.

An eCRF will be provided for each patient. The patient will be identified as per patient-ID only. The eCRF must be completed shortly after each trial visit according to ICH Guideline E6 chapter 4.9.1 and to enable central monitoring of the trial data.

Access to the database will be limited to authorised staff only. Authorisation is granted by the site’s investigator using the trial specific staff signature and delegation log. Based on the staff signature and delegation log access to the eCRF will be granted by the responsible staff at the ZKS Leipzig.

Authorised staff members on site will be able to enter and update data as well as finalise data by electronic signature during the conduct of the trial according to a trial specific concept for documentation. This concept is based on the internal Standard Operating Procedures implemented by the ZKS Leipzig and follows the ICH Guideline E6. All entries and data changes will be tracked automatically including date, time and person who entered/changed information (audit trail). Major correction(s) or major missing data have to be explained.

10.2 Patient File and Source Data

All information required by the protocol and therefor collected during the clinical trial must be recorded by the Investigator or an authorised member of the trial team as source data in the source documentation for the trial (e.g. patient file).

Source data according to ICH Guideline E6 are defined as any information in original records and/or certified copies of original records of clinical findings, observations, or other activities in a clinical trial necessary for the reconstruction and evaluation of the trial. Source data are contained in source documents.

The Source Data Agreement is defining source data and their location for respective CRF entries. It will be filled in at the initiation visit, signed by the investigator or deputy and filed in the trial master file.

In order to confirm the completeness, accuracy and consistency of the data with the data in the source documents the investigator or deputy has to electronically sign each patient's CRF after his/her individual end of trial participation.

10.3 Data Management

For creation of the trial database the EDC tool secuTrial®, developed and distributed by interActive Systems GmbH (iAS), will be used. The database will be validated according to the Standard Operating Procedures (SOPs) of the ZKS Leipzig prior to data capture.

The information entered into the eCRF by the investigator or an authorised member of the trial team is systematically checked for completeness, consistency and plausibility by routines implemented in CDMS such that discrepancies can be dealt with at data entry. Errors and Warnings are listed in a validation report and can be resolved at any time during entry process. On completion of the data entry the site staff flags the eCRF-pages as 'data entry completed' (DEC).

During on-site monitoring or central/statistical monitoring, the monitor or the data manager at the ZKS Leipzig may create a manual query for discrepancies that are identified after DEC. All eCRF pages with queries are marked in the system and a report with all queries listed is available. The site staff is responsible for data correction and can resolve queries directly in the eCRF page.

The ZKS Leipzig will supervise and support the solution of queries and will close all correctly resolved queries. In case a query cannot be solved, the data management staff may close the query in agreement with the trial biometrician.

In case of self-evident corrections (SECs) by data management staff, a list of self-evident corrections or data entry conventions (e.g. for data entry of paper-based life questionnaires) will be available and authorised by the coordinating investigator or the responsible biometrician of the clinical trial. Each trial site will receive a list of performed SECs at the end of the trial.

During the whole course of the trial, a backup of the data is made on a daily basis according to the backup policies of the ZKS Leipzig. Unauthorised access to patient data is prevented by the access concept of the trial database, which is based on a strict hierarchy and role concept. Any change of data (e.g. when data is changed in the database during query management) is recorded automatically via audit trail within the database.

At the end of the trial, once the database has been declared complete and accurate, the database will be locked. Thereafter, any changes to the database are possible only by joint written agreement between sponsor/sponsors authorised representative/coordinating investigator, biometrician and data manager.

10.4 Archiving

All relevant trial documentation (Trial Master File), the electronically stored data, the original CRFs and the final report will be stored for at least 25 years at the university archive Leipzig after the regular or premature end of the trial.

At the investigating sites, the investigators' files, patient identification lists, signed written consent forms, copies of all CRFs and the patients' files will be stored for at least 25 years after the regular or premature end of the trial. Local rules or other legal requirements are to be met if they require longer periods of archiving.

11 SUPERVISION OF THE CLINICAL TRIAL

11.1 Sponsor Statement regarding supervision of trial and access to source data

Herewith the sponsor confirms that the investigators and institutions involved in the clinical trial are to permit clinical trial-related monitoring, audits and regulatory inspections, including provision of direct access to source data and documents.

According to ICH Guideline E6 and EU CTR, the investigator must permit all authorised third parties access to the trial site and the medical records of the trial subjects (source data). These include the clinical trial monitors, auditors and other authorised employees of the sponsor, as well as members of the local or federal authorities. All these persons are sworn to secrecy.

11.2 Monitoring

The ZKS Leipzig will be responsible for trial monitoring. Initiation, regular and close-out visits will be performed in all trial sites. A risk-based monitoring strategy will be implemented, as required by ICH E6 R2 (Chapter 5.0).

During trial conduct, central and statistical monitoring procedures will be combined with on-site monitoring visits in order to achieve high protocol compliance and data quality, as well as to ensure patients' safety and rights. The choosen monitoring strategy depends on the results of the risk analysis done during the protocol development and will be described in the trial specific monitoring plan.

In general, a first monitoring visit at a trial site will be scheduled after the inclusion of the site's first patient, checking protocol compliance and preventing further systematic errors due to misunderstandings. All trial sites will then be visited regularly. The frequency of further on-site monitoring visits will depend on the trial site's recruitment rate and on whether problems have been detected with the site, either by prior on-site visits or by central monitoring.

Prior to every scheduled on-site visit, the monitor will be provided patient synopses summarising the data already available in the database, and indicating possible protocol deviations or inconsistencies. If deemed necessary (e.g. in case of a high number of data

inconsistencies/queries), queries raised by pharmacovigilance and/or from the statistical monitoring will be communicated to the site in due time before the on site visit, to enable a timely processing.

During the visits the monitor will:

- check informed consent forms of all patients enrolled
- perform source data verification of the key data (selected baseline parameters, therapy delivery, serious adverse events, follow-up) in a random sample of the site's patients
- perform targeted source data verification for patients with possible deviations
- discuss open queries raised by data management or drug safety personnel
- check essential parts of the investigator site file (see monitoring plan)
- check source data for AEs or SAEs, which have not been properly reported in the eCRF
- check for major GCP-breaches and/or protocol violations

according to the trial specific monitoring plan.

11.3 Audits

In order to guarantee that the conduct of the trial is in accordance with ICH-GCP and the EU CTR, the sponsor reserves the right to audit selected trial sites. This will be carried out by an unbiased auditor.

The investigator agrees to provide access to all relevant documents for review by the auditor.

11.4 Inspections

According to EU CTR, inspections of the trial sites may be performed by the local or federal authorities at any time during or after completion of the trial. The investigator agrees to provide access to all relevant documents for review by the inspectors.

12 DATA PROTECTION

12.1 Data protection and Confidentiality

The sponsor, together with ZKS Leipzig and the trial sites, is responsible for the implementation and data processing in accordance with Article 4(7) of the EU Data Protection Basic Regulation 2016/679 in this trial. The ZKS Leipzig is responsible for implementation of procedures for data collection, storage, protection, retention and destruction. The ZKS Leipzig has implemented a data safety and security concept according to the requirements of the German Federal Office for Information Security (www.bsi.bund.de).

All data will be initially collected by investigators in the recruiting trial sites. Together with information on the trial, eligible patients will be informed about data capture, transmission, analysis processes and their rights according to the General Data Protection Regulation (GDPR). Once a patient is eligible and has given his/her informed consent to trial participation and data collection, the investigator will assign the patient a unique patient identification code. Patient identification code lists will be generated in advance by ZKS Leipzig and forwarded to

the recruiting sites. These lists are part of the investigator site file and remain at the recruiting site. These lists are the only documents that allow for re-identification of the patients.

All clinical data entered by the investigators (or their designated staff) into eCRFs will be recorded in a pseudonymized form (i.e. without reference to the patient's name and date of birth) exclusively using the patient's identification code.

Clinical monitors appointed by ZKS Leipzig will regularly visit the recruiting sites and verify the informed consent forms. Data will only be used for analysis after it has been verified by monitors that the patient has unambiguously given his or her consent for trial participation as well as for data capture, transmission and analysis. The patients are informed of this fact and agree to the procedure with the patient information/informed consent.

In the event of withdrawal of consent, the necessity for storing data will be evaluated. While the General Data Protection Regulation (EU) 2016/679 strengthens personal data protection rights, encompassing the right to access, rectification and withdrawal of data, it also specifies the situations when restriction on those rights may be imposed. The withdrawal of informed consent should not affect the results of activities already carried out, such as the storage and use of data obtained on the basis of informed consent before.

12.1.1 Measures to ensure data confidentiality

In principle, targeted criminal cyberattacks can never be completely avoided or ruled out. Within the means available to an academic institution, the following measures are planned to prevent such a cyberattack:

- Comprehensive QM system of the IT infrastructure based on SOPs
- Active business continuity plan
- Strict separation between IT infrastructures that process personal data and structures that do not
- Central user administration with strict access rules, incl. additional authentication when using different areas of the infrastructure
- Sealing off the web servers in the demilitarised zone (DMZ) of the infrastructure
- Strict requirements for the maintenance/updating of the systems, including requirements for the prompt installation of security-relevant patches and updates
- Strict guidelines for mobile working, including guidelines for the use of external storage media

12.1.2 Measures in case of data security breaches

Emergency and recovery plans are in place as part of the business continuity of ZKS Leipzig. In the event of a cyberattack, there is a switch to emergency operation that makes it possible to maintain study operations. This includes measures for the randomisation and registration of study participants, the recording and transfer of security-relevant information from the studies and the switch to paper-based documentation of patient data in the event that eCRF is used.

12.2 Anonymisation of Data after the end of Archiving

Not applicable.

13 ADMINISTRATIVE AGREEMENTS

The clinical trial described here will be conducted and analysed in accordance with EU CTR

and ICH Guideline E6 (Good Clinical Practice - GCP).

13.1 Adherence to the Protocol

Protocol violations are all deviations from the procedures outlined in this document. After a patient has been enrolled, it is the investigator's responsibility to avoid protocol violations in order to obtain unbiased data for the trial.

Protocol violations are deemed to be major if they lead to exclusion from the PPS (see 9.5.1) Furthermore, a risk analysis performed before and during trial implementation will be further detailed in separate documents belonging to the risk assessment/monitoring plan and may identify further relevant protocol violations. This list can be augmented in the course of the trial. Major protocol violations will be reported to the ZKS Leipzig, which will inform the sponsor.

All protocol violations will be documented and discussed with the responsible biometrician before closing the data base and carrying out the statistical analysis.

The investigator must ensure that the recorded data are documented as per protocol. Minor variations are inevitable in clinical routine, but must be documented together with a justification.

13.2 Serious Breaches

According to EU CTR Art. 52: “The sponsor shall notify the Member States concerned about a serious breach of this Regulation or of the version of the protocol applicable at the time of the breach through the EU portal without undue delay but not later than seven days of becoming aware of that breach.”

Serious breach means a breach likely to affect to a significant degree the safety and rights of a subject or the reliability and robustness of the data generated in the clinical trial.

If an investigator or other trial personnel is aware of the occurrence of a (possible) serious breach, they inform the study team at ZKS Leipzig immediately via the trial-specific email address panem@zks.uni-leipzig.de. Further information about the reporting obligations will be described in a study specific working instruction in the investigator site file.

13.3 Funding and Insurance

The PANEM-Trial is funded by:

Repha GmbH Biologische Arzneimittel, Alt-Godshorn 87, 30855 Langenhagen

The sponsor is responsible for the provision of insurance or indemnity to cover potential harm done to the patients due to the trial participation according to the national requirements and the corresponding national laws. All trial participants will be insured during trial participation by a volunteers' trial insurance to the medical faculty of the Leipzig University:

Policy number: 28-138971-03302 (03/2022)

Policy holder: HDI Global SE, Eisenbahnstraße 1-3, 04315 Leipzig, Germany

Maximum sum insured: 500,000.00 €/patient

At each trial site, a copy of the policy and the general policy conditions will be filed in the ISF. The patient will receive a copy of the general policy conditions.

13.4 Publication Policy and Registration

The results of this trial will be submitted for publication in a peer-reviewed, international English-language journal of appropriate aim and scope. Accordingly, the clinical trial will be registered at clinicaltrials.gov before recruitment starts. According to the results of main and concomitant studies, the results may be submitted in separate or combined manuscripts; decisions about the form and scope of individual manuscripts will be discussed among all persons participating in the design, conduct and analysis of the trial who qualify for authorship. The coordinating investigator together with the biometrician(s) is responsible for drafting and circulating manuscripts and for discussing and handling requests by co-authors or/and sponsors to edit the text.

The authorship will follow the criteria for authorship developed by the International Committee of Medical Journal Editors (ICMJE), including those that distinguish authors from other contributors.

The ICMJE recommends that authorship be based on the following 4 criteria:

- Substantial contributions to the conception or design of the work; or the acquisition, analysis, or interpretation of data for the work; AND
- Drafting the work or revising it critically for important intellectual content; AND
- Final approval of the version to be published; AND
- Agreement to be accountable for all aspects of the work in ensuring that questions related to the accuracy or integrity of any part of the work are appropriately investigated and resolved.

All those designated as authors should meet all four criteria for authorship, and all who meet the four criteria should be identified as authors. Those who do not meet all four criteria will be acknowledged in the manuscript.

Each site that contributes at least 3 patients to the FAS and has complete documentation for these patients can list a single author. Each site that contributes at least 8 patients to the FAS and has complete documentation for these patients can list two authors. The first and senior authorships are reserved for the team of the coordinating investigator and the order of authors from recruiting sites depends on the number of patients recruited from the respective site.

The scientific use of data resulting from this trial by local trial sites is ruled by the site contracts between the sponsor and the local trial sites. Generally, sites might use data for own scientific questions (independent from the questions discussed in this trial protocol) and publication after consultation with the sponsor.

13.5 Data Sharing Statement

According to the recommendations on data sharing by the International Committee of Medical Journal Editors (ICMJE) data resulting from the PANEM-trial will be made available to the scientific community as follows:

After publication of the major results and upon reasonable request from researchers performing an individual patient data meta-analysis, individual patient data that underlie published results will be shared after de-identification. This requires approval by the local

Institutional Review Board (IRB) of the researcher requesting the data along with public registration of the meta-analysis.

Summary statistics that go beyond the scope of published material will be made available to researchers for meta-analysis upon reasonable request and if the necessary data analysis is not unduly time-consuming. Together with publication of the main results, the trial protocol in full will be made publically available as well as the statistical analysis plan

14 PROTOCOL SIGNATURES

Confirmation of the Final Protocol

I hereby certify that this is the final version of the protocol:

Authorised representative of the sponsor:	______________________<br>Date	______________________<br>Signature

15 PROTOCOL AGREEMENT

(vom (Haupt-) ***Prüfer*** *des jeweiligen Zentrums vor Eintritt des Zentrums in die Studie bzw. vor Behandlung von Studienteilnehmern zu unterzeichnen)*

Herewith I declare that I have read and understood the present protocol and agree to take into account and follow each part of it. I will ensure that all trial patients enrolled in this trial site will be treated, observed and documented in accordance with this protocol. I will ensure that all investigators and other persons assisting with the trial under my supervision are adequately informed about the protocol, the investigational product and their duties.

I further declare that I do not have any financial and other competing interests in this trial.

Centre-ID ______ - __/__/__/

Address trial site (stamp)

Date	Signature *(Principal)* Investigator

16 APPENDIX

16.1 Classification of Adverse Events

16.1.1 Degree of Seriousness

The seriousness of an Adverse Event will be determined in accordance with the definitions in 8.1 and 8.4.

16.1.2 Assessment of Intensity/Severity

The assessment of the intensity accords with CTCAE V5.0

Mild Adverse Event	• asymptomatic or mild symptoms;<br>• clinical or diagnostic observations only;<br>• intervention not indicated.
Moderate Adverse Event	• minimal, local or noninvasive intervention indicated;<br>• limiting age-appropriate instrumental ADL*[14].
Severe Adverse Event	• medically significant but not immediately life-threatening;<br>• hospitalisation or prolongation of hospitalisation indicated;<br>• disabling;<br>• limiting self care ADL**
Life-threatening Adverse Event	• Life-threatening consequences;<br>• urgent intervention indicated
Death related to Adverse Event	

16.1.3 Determining the Causal Relationship

The investigator/ the deputy or the authorised medical staff must assess whether or not the Adverse Event is causally related to the administration of the trial medication. The following classification is to be used.

- Reasonable possibility

[14] **Activities of Daily Living (ADL):**
*Instrumental ADL refer to preparing meals, shopping for groceries or clothes, using the telephone, managing money, etc.
**Self care ADL refer to bathing, dressing and undressing, feeding self, using the toilet, taking medications, and not bedridden.

- No reasonable possibility

A reasonable possibility exists, if one of the following WHO-UMC criteria is met:

- occurring in a plausible time relationship to drug administration, and which cannot be explained by concurrent disease or other drugs or chemicals. The response to withdrawal of the drug (dechallenge) should be clinically plausible. The event must be definitive pharmacologically or phenomenologically, using a satisfactory rechallenge procedure if necessary.
- with a reasonable time sequence to administration of the drug, unlikely to be attributed to concurrent disease or other drugs or chemicals, and which follows a clinically reasonable response on withdrawal (dechallenge). Rechallenge information is not required to fulfil this definition.
- with a reasonable time sequence to administration of the drug, but which could also be explained by concurrent disease or other drugs or chemicals. Information on drug withdrawal may be lacking or unclear.
- more data is essential for a proper assessment or the additional data are under examination
- cannot be judged because information is insufficient or contradictory, and which cannot be supplemented or verified

No reasonable possibility exists, if the following WHO-UMC criterion is met:

- with a temporal relationship to drug administration which makes a causal relationship improbable, and in which other drugs, chemicals or underlying disease provide plausible explanations.

16.1.4 Expected/Unexpected

Adverse Events are unexpected if they do not occur in the manner or with the intensity described in the SmPC/Investigator’s Brochure (see investigator’s files).

16.1.5 Outcome of an Adverse Event

The outcome of an Adverse Event is classified as follows:

- recovered/resolved
- recovering/resolving
- not recovered/not resolved
- recovered/resolved with sequel
- fatal*
- unknown

*Note: A patient’s death is not in itself an event, but the consequence of one. The event that led to the patient’s death must be documented completely and reported even if death occurs four weeks after stopping medication and independent of whether or not there is a relation to the therapy or not.

16.2 Acronyms

AE	Adverse Event
AGI	Alpha-Glucosidase Inhibitor
AMG	Arzneimittelgesetz (= German Medicinal Products Act)
ASR	Annual Safety Report
BfArM	Bundesinstitut für Arzneimittel und Medizinprodukte (German Competent Authority)
BMI	Body Mass Index
CA	Competent Authority
DEC	Data Entry Completed
eCRF	electronic Case Report Form
EU CTR	Regulation (EU) 536/2014
FE-1	Faecal Elastase-1
GCP	Good Clinical Practice
GCP-V	GCP-Verordnung
GMP	Good Manufacturing Practice
ICH	International Council for Harmonisation
IMP	Investigator Medicinal Product
IMPD	Investigator Medicinal Product Dossier
ITT	Intention To Treat
LPLV	Last Patient Last Visit
MCID	Minimal Clinically Important Difference
PEI	Paul-Ehrlich-Institut (Agency of the German Federal Ministry of Health. Its research and control activities promote the quality, efficacy and safety of biological medicinal products.)
PPS	Per Protocol Set
QoL	Quality of Life
RCT	Randomized Controlled Trial
SAE	Serious Adverse Event
SAP	Statistical Analysis Plan
SAR	Serious Adverse Reaction
Scr	Screening
SD	Standard Deviation
SEC	Self-Evident Correction
SmPC	Summary of medicinal Product Characteristics

SOP	Standard Operating Procedure
SUSAR	Suspected Unexpected Serious Adverse Reaction
SV	Survival Visits
WHO-UMC	World Health Organization – Uppsala Monitoring Centre
ZKS Leipzig	Zentrum für klinische Studien Leipzig

16.3 Labelling

On the immediate and the outer packaging:

Sponsor: Universität Leipzig
Prof. Dr. med. A. Hoffmeister; Tel: +49 341/9712240
EU CT-Nr.: 2022-501944-14-00 Prüfplancode: PANEM

Rizolipase (Lipase aus R. oryzae) entspr. 7.000 FIP-Einheiten,
Protease (mind. 54 FIP-Einheiten) und Amylase (mind. 700 FIP-Einheiten) aus A. oryzae
ODER Placebo
Zur Klinischen Prüfung bestimmt!
Für Kinder unzugänglich aufbewahren
Nicht verbrauchte Prüfpräparate bitte aufbewahren
MedKit-Nr: AXY
Ch.-B.: 22A09 12, verwendbar bis: **MM/JJJJ**
Anwendung nach Vorgabe Ihres Arztes!
100 Kapseln, zur oralen Einnahme bestimmt

16.4 Template trial protocol

This trial protocol was written based on a template by the ZKS Leipzig based on the SOPs of the ZKS Leipzig.

The used template version is: EUCTR draft0.2 from 2022-05-17

17 REFERENCES

1. Hutchinson DR. Indexed ICH GCP guidelines with integrated addendum E6(R2), Step 4, November 2016, Integrated addendum to ICH E6(R1) : Guideline for good clinical practice E6(R2) : current step 4 version, dated 9 November 2016. Chobham, Surrey: Canary, 2016.
2. Thrumurthy SG, Chaudry MA, Hochhauser D, Mughal M. The diagnosis and management of gastric cancer. BMJ 2013;347:f6367.
3. Griffin SM, Kamarajah SK, Navidi M, et al. Evolution of gastrectomy for cancer over 30-years: Changes in presentation, management, and outcomes. Surgery 2021.
4. Fukuda Y, Yamamoto K, Hirao M, et al. Prevalence of Malnutrition Among Gastric Cancer Patients Undergoing Gastrectomy and Optimal Preoperative Nutritional Support for Preventing Surgical Site Infections. Ann Surg Oncol 2015;22 Suppl 3:S778-85.
5. Armbrecht U, Lundell L, Stockbrügger RW. The benefit of pancreatic enzyme substitution after total gastrectomy. Aliment Pharmacol Ther 1988;2:493–500.
6. Brägelmann R, Armbrecht U, Rosemeyer D, Schneider B, Zilly W, Stockbrügger RW. The effect of pancreatic enzyme supplementation in patients with steatorrhoea after total gastrectomy. Eur J Gastroenterol Hepatol 1999;11:231–7.
7. Catarci M, Berlanda M, Grassi GB, Masedu F, Guadagni S. Pancreatic enzyme supplementation after gastrectomy for gastric cancer: a randomized controlled trial. Gastric Cancer 2018;21:542–51.
8. Straatman J, Wiegel J, van der Wielen N, Jansma EP, Cuesta MA, van der Peet DL. Systematic Review of Exocrine Pancreatic Insufficiency after Gastrectomy for Cancer. Dig Surg 2017;34:364–70.
9. Deutsche Krebsgesellschaft e.V., Zertifizierungskommission Magenkrebszentren, Mayerle J, et al. Qualitätsindikatoren Magenkrebs 2021, Jahresbericht der zertifizierten Magenkrebszentren 2021.
10. Lordick F, Al-Batran S-E, Arnold D, et al. German, Austrian, and Swiss guidelines for systemic treatment of gastric cancer. Gastric Cancer 2023;27:6–18.
11. Crew KD, Neugut AI. Epidemiology of gastric cancer. World J Gastroenterol 2006;12:354–62.
12. Aaronson NK, Ahmedzai S, Bergman B, et al. The European Organization for Research and Treatment of Cancer QLQ-C30: a quality-of-life instrument for use in international clinical trials in oncology. J Natl Cancer Inst 1993;85:365–76.
13. Waldmann A, Schubert D, Katalinic A. Normative data of the EORTC QLQ-C30 for the German population: a population-based survey. PLoS One 2013;8:e74149.
14. Avery K, Hughes R, McNair A, Alderson D, Barham P, Blazeby J. Health-related quality of life and survival in the 2 years after surgery for gastric cancer. Eur J Surg Oncol 2010;36:148–54.
15. Climent M, Munarriz M, Blazeby JM, et al. Weight loss and quality of life in patients surviving 2 years after gastric cancer resection. Eur J Surg Oncol 2017;43:1337–43.
16. Lee I, Oh Y, Park S-H, Kwon Y, Park S. Postoperative nutritional outcomes and quality of life-related complications of proximal versus total gastrectomy for upper-third early gastric cancer: a meta-analysis. Sci Rep 2020;10:21460.
17. Blazeby JM, Conroy T, Bottomley A, et al. Clinical and psychometric validation of a questionnaire module, the EORTC QLQ-STO 22, to assess quality of life in patients with gastric cancer. Eur J Cancer 2004;40:2260–8.

18. Kobayashi D, Kodera Y, Fujiwara M, Koike M, Nakayama G, Nakao A. Assessment of quality of life after gastrectomy using EORTC QLQ-C30 and STO22. World J Surg 2011;35:357–64.
19. Vickery C, Blazeby J, Conroy T, et al. Development of an EORTC disease-specific quality of life module for use in patients with gastric cancer. European Journal of Cancer 2001;37:966–71.
20. Martin L, Lagergren P. Long-term weight change after oesophageal cancer surgery. Br J Surg 2009;96:1308–14.
21. Aoyama T, Maezawa Y, Yoshikawa T, et al. Comparison of Weight and Body Composition After Gastrectomy Between Elderly and Non-elderly Patients With Gastric Cancer. In Vivo 2019;33:221–7.
22. Davis JL, Selby LV, Chou JF, et al. Patterns and Predictors of Weight Loss After Gastrectomy for Cancer. Ann Surg Oncol 2016;23:1639–45.
23. An JY, Youn HG, Choi MG, Noh JH, Sohn TS, Kim S. The difficult choice between total and proximal gastrectomy in proximal early gastric cancer. Am J Surg 2008;196:587–91.
24. Ida S, Hiki N, Cho H, et al. Randomized clinical trial comparing standard diet with perioperative oral immunonutrition in total gastrectomy for gastric cancer. Br J Surg 2017;104:377–83.
25. Domínguez-Muñoz JE, Iglesias-García J, Iglesias-Rey M, Figueiras A, Vilariño-Insua M. Effect of the administration schedule on the therapeutic efficacy of oral pancreatic enzyme supplements in patients with exocrine pancreatic insufficiency: a randomized, three-way crossover study. Aliment Pharmacol Ther 2005;21:993–1000.
26. Capurso G, Traini M, Piciucchi M, Signoretti M, Arcidiacono PG. Exocrine pancreatic insufficiency: prevalence, diagnosis, and management. Clin Exp Gastroenterol 2019;12:129–39.
27. Raman S, Ding K, Chow E, et al. Minimal clinically important differences in the EORTC QLQ-C30 and brief pain inventory in patients undergoing re-irradiation for painful bone metastases. Qual Life Res 2018;27:1089–98.
28. Wong E, Zhang L, Kerba M, et al. Minimal clinically important differences in the EORTC QLQ-BN20 in patients with brain metastases. Support Care Cancer 2015;23:2731–7.
29. Zeng L, Chow E, Zhang L, et al. An international prospective study establishing minimal clinically important differences in the EORTC QLQ-BM22 and QLQ-C30 in cancer patients with bone metastases. Support Care Cancer 2012;20:3307–13.
30. Yu J, Huang C, Sun Y, et al. Effect of Laparoscopic vs Open Distal Gastrectomy on 3-Year Disease-Free Survival in Patients With Locally Advanced Gastric Cancer: The CLASS-01 Randomized Clinical Trial. JAMA 2019;321:1983–92.
31. Wu CW, Lo SS, Shen KH, Hsieh MC, Lui WY, P'eng FK. Surgical mortality, survival, and quality of life after resection for gastric cancer in the elderly. World J Surg 2000;24:465–72.
32. Liu Z, Feng F, Guo M, et al. Distal gastrectomy versus total gastrectomy for distal gastric cancer. Medicine (Baltimore) 2017;96:e6003.
33. Tian Q, Qin L, Zhu W, Xiong S, Wu B. Analysis of factors contributing to postoperative body weight change in patients with gastric cancer: based on generalized estimation equation. PeerJ 2020;8:e9390.
