# Supplementary material for: Protocol of the PANEM trial: A randomized, phase III, parallel-arm, placebo-controlled, double-blind, multi-center superiority trial comparing PANcreatic EnzyMes versus placebo in adults after gastrectomy
Source: PLoS One. 2026 Sep 22;21(9):e0358177. doi: 10.1371/journal.pone.0358177 (PMC13596798; doi:10.1371/journal.pone.0358177)
Supplement: Peer review history

# Peer review: Decision Letter 0

**DOI:** 10.1371/journal.pone.0358177.r001

**Authors:** M Saad Saumtally

30 Mar 2026

Dear Dr. Thomas,

Thank you for submitting your manuscript to PLOS ONE. After careful consideration, we feel that it has merit but does not fully meet PLOS ONE’s publication criteria as it currently stands. Therefore, we invite you to submit a revised version of the manuscript that addresses the points raised during the review process.

Please submit your revised manuscript by May 14 2026 11:59PM. If you will need more time than this to complete your revisions, please reply to this message or contact the journal office at plosone@plos.org. When you're ready to submit your revision, log on to https://www.editorialmanager.com/pone/ and select the 'Submissions Needing Revision' folder to locate your manuscript file.

- A letter that responds to each point raised by the academic editor and reviewer(s). You should upload this letter as a separate file labeled 'Response to Reviewers'.
- A marked-up copy of your manuscript that highlights changes made to the original version. You should upload this as a separate file labeled 'Revised Manuscript with Track Changes'.
- An unmarked version of your revised paper without tracked changes. You should upload this as a separate file labeled 'Manuscript'.

If you would like to make changes to your financial disclosure, please include your updated statement in your cover letter. Guidelines for resubmitting your figure files are available below the reviewer comments at the end of this letter.

If applicable, we recommend that you deposit your laboratory protocols in protocols.io to enhance the reproducibility of your results. Protocols.io assigns your protocol its own identifier (DOI) so that it can be cited independently in the future. For instructions see: https://journals.plos.org/plosone/s/submission-guidelines#loc-laboratory-protocols. Additionally, PLOS ONE offers an option for publishing peer-reviewed Lab Protocol articles, which describe protocols hosted on protocols.io. Read more information on sharing protocols at https://plos.org/protocols?utm_medium=editorial-email&utm_source=authorletters&utm_campaign=protocols.

As the corresponding author, your ORCID iD is verified in the submission system and will appear in the published article. PLOS supports the use of ORCID, and we encourage all coauthors to register for an ORCID iD and use it as well. Please encourage your coauthors to verify their ORCID iD within the submission system before final acceptance, as unverified ORCID iDs will not appear in the published article. *Only*  the individual author can complete the verification step; PLOS staff *cannot*  verify ORCID iDs on behalf of authors.

We look forward to receiving your revised manuscript.

Kind regards,

M Saad Saumtally

Academic Editor

PLOS One

Journal Requirements:

When submitting your revision, we need you to address these additional requirements.

1. Please ensure that your manuscript meets PLOS ONE's style requirements, including those for file naming. The PLOS ONE style templates can be found at

https://journals.plos.org/plosone/s/file?id=wjVg/PLOSOne_formatting_sample_main_body.pdf and

https://journals.plos.org/plosone/s/file?id=ba62/PLOSOne_formatting_sample_title_authors_affiliations.pdf

2. PLOS requires an ORCID iD for the corresponding author in Editorial Manager on papers submitted after December 6th, 2016. Please ensure that you have an ORCID iD and that it is validated in Editorial Manager. To do this, go to ‘Update my Information’ (in the upper left-hand corner of the main menu), and click on the Fetch/Validate link next to the ORCID field. This will take you to the ORCID site and allow you to create a new iD or authenticate a pre-existing iD in Editorial Manager.

3. We note that the grant information you provided in the ‘Funding Information’ and ‘Financial Disclosure’ sections do not match.

When you resubmit, please ensure that you provide the correct grant numbers for the awards you received for your study in the ‘Funding Information’ section.

4. Thank you for stating the following financial disclosure:

The PANEM trial is funded by Repha GmbH Biologische Arzneimittel, Alt-Godshorn.

The funding body did not participate in designing the trial or analyzing data.

Please state what role the funders took in the study. If the funders had no role, please state: "The funders had no role in study design, data collection and analysis, decision to publish, or preparation of the manuscript."

If this statement is not correct you must amend it as needed.

Please include this amended Role of Funder statement in your cover letter; we will change the online submission form on your behalf.

5. We note that you have indicated that there are restrictions to data sharing for this study. For studies involving human research participant data or other sensitive data, we encourage authors to share de-identified or anonymized data. However, when data cannot be publicly shared for ethical reasons, we allow authors to make their data sets available upon request. For information on unacceptable data access restrictions, please see http://journals.plos.org/plosone/s/data-availability#loc-unacceptable-data-access-restrictions.

Before we proceed with your manuscript, please address the following prompts:

a) If there are ethical or legal restrictions on sharing a de-identified data set, please explain them in detail (e.g., data contain potentially identifying or sensitive patient information, data are owned by a third-party organization, etc.) and who has imposed them (e.g., a Research Ethics Committee or Institutional Review Board, etc.). Please also provide contact information for a data access committee, ethics committee, or other institutional body to which data requests may be sent.

b) If there are no restrictions, please upload the minimal anonymized data set necessary to replicate your study findings to a stable, public repository and provide us with the relevant URLs, DOIs, or accession numbers. Please see http://www.bmj.com/content/340/bmj.c181.long for guidelines on how to de-identify and prepare clinical data for publication. For a list of recommended repositories, please see https://journals.plos.org/plosone/s/recommended-repositories. You also have the option of uploading the data as Supporting Information files, but we would recommend depositing data directly to a data repository if possible.

Please update your Data Availability statement in the submission form accordingly.

6. When completing the data availability statement of the submission form, you indicated that you will make your data available on acceptance. We strongly recommend all authors decide on a data sharing plan before acceptance, as the process can be lengthy and hold up publication timelines. Please note that, though access restrictions are acceptable now, your entire data will need to be made freely accessible if your manuscript is accepted for publication. This policy applies to all data except where public deposition would breach compliance with the protocol approved by your research ethics board. If you are unable to adhere to our open data policy, please kindly revise your statement to explain your reasoning and we will seek the editor's input on an exemption. Please be assured that, once you have provided your new statement, the assessment of your exemption will not hold up the peer review process.

7. Please amend the manuscript submission data (via Edit Submission) to include authors J. Hipp, T. Bley, J. Sporn, F. Peters, P. Téoule, and B. Emrullah.

8. Please include captions for your Supporting Information files at the end of your manuscript, and update any in-text citations to match accordingly. Please see our Supporting Information guidelines for more information: http://journals.plos.org/plosone/s/supporting-information.

9. If the reviewer comments include a recommendation to cite specific previously published works, please review and evaluate these publications to determine whether they are relevant and should be cited. There is no requirement to cite these works unless the editor has indicated otherwise.

[Note: HTML markup is below. Please do not edit.]

Reviewers' comments:

Reviewer's Responses to Questions

**Comments to the Author**

1. Does the manuscript provide a valid rationale for the proposed study, with clearly identified and justified research questions?

Reviewer #1: Partly

Reviewer #2: Yes

Reviewer #3: Yes

**********

2. Is the protocol technically sound and planned in a manner that will lead to a meaningful outcome and allow testing the stated hypotheses?

Reviewer #1: Yes

Reviewer #2: Yes

Reviewer #3: Yes

**********

3. Is the methodology feasible and described in sufficient detail to allow the work to be replicable?

Reviewer #1: Yes

Reviewer #2: No

Reviewer #3: Yes

**********

4. Have the authors described where all data underlying the findings will be made available when the study is complete?

The PLOS Data policy

Reviewer #1: Yes

Reviewer #2: Yes

Reviewer #3: Yes

**********

5. Is the manuscript presented in an intelligible fashion and written in standard English?

Reviewer #1: Yes

Reviewer #2: Yes

Reviewer #3: Yes

**********

Please use the space provided to explain your answers to the questions above and, if applicable, provide comments about issues authors must address before this protocol can be accepted for publication. You may also include additional comments for the author, including concerns about research or publication ethics.

You may also provide optional suggestions and comments to authors that they might find helpful in planning their study.

Reviewer #1: As the statistical reviewer I will focus on methods and reporting.

Major

1) The power calculations do not account for the multicentre nature of the trial. Currently the ICC is expected to be zero. is that a reasonable assumption?

2) how are missing data implicitly dealt by the mixed effects model? as currently described, it is a complete case analysis, unless there is a Bayesian element to the model and there is data augmentation at play. So some clarity is needed and ideally some information on the use of multiple imputation techniques, if relevant, as I would expect them to be.

3) if some centres recruit very few patients they may need to be excluded from analyses as a sensitivity, if their inclusion imbalances the estimated random intercepts for centre.

Minor

1) the proportional hazards assumption in the Cox model needs to be evaluated.

2) the gold standard for anlaysing continuous outcomes in time to even data where mortality is a potential concern, is joint models - discuss as a limitation if you don't conduct, or as a sensitivitiy. Excluding deaths creates immortal time bias and deaths need to be closely monitored and reported to ensure balance across arms.

Reviewer #2: Hello, this is an interesting and important area of research that has the potential to impact thousands of patients lives.

Please see comments below.

There are several areas where clarification of the study protocol and methods and well as justification for decisions are required. I hope these comments are useful.

Lines 78-80 – please can you check the wording on this? as 5yr mortality rates for gastric cancer are around 24%. Do you mean gastric cancer deaths as a whole?

Lines: 82-84 - ? reference.

Lines 124 – 129: can this be re-worded to include the specific aim of the trial for clarity

Line: 136 – ‘approximately’ rather than ‘about’

Lines: 156 – 160 – can there be further clarification with the inclusion criteria? Is this all patients – cancer/bariatric surgery/emergency surgery e.g., trauma, GOO etc,

Table 1 – you have stated ‘key’ inclusion criteria – is this all exclusion criteria or just the main ones? Is just the main ones please list all exclusion criteria

Lines 163 – 167 – please review for clarity. How long post gastrectomy can patients be consented and therefore started on trial? You have stated ‘no later than three days after beginning oral nutrition’ – is this for consenting or starting PERT/placebo? Do you have plans for what happens if oral nutrition is delayed? E.g., patients require PN post surgery – can they still be included?

Lines 170 – 177: Can the units of lipase (lipase units/kg) be stated for the PERT

Lines 182 – 188: Who is going to be advising patients to increase/decrease dosage? Are they blind to the intervention? Is standardised advice being given regarding the timings/correct methos of taking PERT/placebo? Please state if this is / isn’t the case, with justification.

What is the justification for only increasing PERT at 3 weeks?

You have stated that the exclusion criteria include ‘indication for PERT’. Please state what happens if someone is experiencing steatorrhea/malabsorption symptoms post-op and once consented to the trial? If they are in the control group are they withdrawn from the trial? As it is not ethical to not provide them with PERT? – noted you have an SAE section – this could be clarified in either section.

Lines 199 – 201 – you’ve stated ‘disease related laboratory measurements’ here, but ‘laboratory measurements related to malnutrition and metabolism’ in the abstract. Please change the abstract to match the body of the article.

Line 210: again, reads as nutrition related blood parameters, please re-word e.g., disease related blood parameters (as Hb, alb, total protein are not nutrition-related measures)

Line 216 – how is glucose measured? Fasting? Random?

Lines 209 – 218 – noted Fig 1 / 2 are provided, but a brief summary of when measures will be taken would improve clarity e.g., baseline, week 3, month 3, month 6, etc..

Fig 1 – states screening is max 10 days before gastrectomy until day ?? (image is unclear) – this does not match text (which states ‘not more than 30 days’), when is the final end point – fig1 states every 6m after 6m – for how long? Please state/clarify.

Lines: 233 – 234 – How are eating habits screened? Screening tool? Food diary etc.

Lines: 235 – 237 – Please review – states medication will be started at randomisation, but that is incorrect, no? as meds started with oral nutrition?

245 -246 – is this the only way that medication compliance is being checked? Is this not asked during the telephone visits/reviews? What is the evidence for the reliability of empty medication bottles being a proxy for medications consumed? Please justify.

Lines 250 – 251 – please review for clarity – ‘if an adverse effect occurs’? – any examples?

Lines 293 – 299 – details regarding randomisation. Please can additional information be provided regarding randomisation, in particular in relation to the distribution of the PERT/placebo?

Reviewer #3: Many thanks for sending the protocol for an Investigator-led, Industry-funded RCT of PERT after gastrectomy. Is it well-written and i located the information i would expect. Overall, we need more data in this space and I support the study. I had a few comments which could improve the protocol if addressed:

1. I support the use of the EORTC-QLQ-C30 for the power calculation / primary outcome given it is well-validated in western populations. In a few places it is described as disease-specific (e.g. abstract, methods), which if i have understood things correctly is not accurate. Secondly, there is an opportunity to obtain more grandular data about the benefit of PERT with a gastric-specific assessment, like PGSAS-45. Can a gastrectomy-specific QOL score be added as secondary outcome?

2. I would suggest expanding the exclusion critieria to patients who have had distal GI surgery, or currently have an active distal GI problem. For example patients who have already lost colon or the ileocaecal valve will already have very different baseline QOL compared to others, and certain subgroups e.g. FAP often have gastric surgery after colonic surgery. The effect of losing the pylorus will also be different in patients with distal pathology/surgery. How to handle distal stomas.

3. Participant initiation on the protocol is vague and should be more precisley defined. Is the treatment to be commenced as oral intake is commenced? Is the protocol for initiating oral intake uniform between centres? Is the plan to give the treatment only once food initiated? How are complications like leaks to be handled, and is there a safety concern to the anastomosis, of giving oral proteases in the immediate post-operative period?

**********

what does this mean?). If published, this will include your full peer review and any attached files.

If you choose “no”, your identity will remain anonymous but your review may still be made public.

**Do you want your identity to be public for this peer review?**  For information about this choice, including consent withdrawal, please see our Privacy Policy

Reviewer #1: No

Reviewer #2: No

Reviewer #3: No

**********

[NOTE: If reviewer comments were submitted as an attachment file, they will be attached to this email and accessible via the submission site. Please log into your account, locate the manuscript record, and check for the action link "View Attachments". If this link does not appear, there are no attachment files.]

To ensure your figures meet our technical requirements, please review our figure guidelines: https://journals.plos.org/plosone/s/figures

You may also use PLOS’s free figure tool, NAAS, to help you prepare publication quality figures: https://journals.plos.org/plosone/s/figures#loc-tools-for-figure-preparation.

NAAS will assess whether your figures meet our technical requirements by comparing each figure against our figure specifications.

# Author comment: Author response to Decision Letter 1

**DOI:** 10.1371/journal.pone.0358177.r002

12 Jun 2026

1. Please ensure that your manuscript meets PLOS ONE's style requirements, including those for file naming. The PLOS ONE style templates can be found at

https://journals.plos.org/plosone/s/file?id=wjVg/PLOSOne_formatting_sample_main_body.pdf and

https://journals.plos.org/plosone/s/file?id=ba62/PLOSOne_formatting_sample_title_authors_affiliations.pdf

We adapted our manuscript according to the style requirements.

2. PLOS requires an ORCID iD for the corresponding author in Editorial Manager on papers submitted after December 6th, 2016. Please ensure that you have an ORCID iD and that it is validated in Editorial Manager. To do this, go to ‘Update my Information’ (in the upper left-hand corner of the main menu), and click on the Fetch/Validate link next to the ORCID field. This will take you to the ORCID site and allow you to create a new iD or authenticate a pre-existing iD in Editorial Manager.

We have included the corresponding ORCID ID.

3. We note that the grant information you provided in the ‘Funding Information’ and ‘Financial Disclosure’ sections do not match.

When you resubmit, please ensure that you provide the correct grant numbers for the awards you received for your study in the ‘Funding Information’ section.

We have changed the „Competing interests” to read as follows: “The authors declare that they have no competing interests beyond the financing of this trial. No author receives payment in kind or otherwise in any form from Repha GmbH.” There was no grant number associated with the funding.

4. Thank you for stating the following financial disclosure:

The PANEM trial is funded by Repha GmbH Biologische Arzneimittel, Alt-Godshorn.

The funding body did not participate in designing the trial or analyzing data.

Please state what role the funders took in the study. If the funders had no role, please state: "The funders had no role in study design, data collection and analysis, decision to publish, or preparation of the manuscript."

We have adopted this formulation, adapting it to our situation: “The funder has no role in study design, data collection or preparation of this manuscript or decision to publish. Furthermore, the funder will have no role in the analysis, decision to publish, or preparation of the manuscript presenting the results of the trial.”

If this statement is not correct you must amend it as needed.

Please include this amended Role of Funder statement in your cover letter; we will change the online submission form on your behalf.

5. We note that you have indicated that there are restrictions to data sharing for this study. For studies involving human research participant data or other sensitive data, we encourage authors to share de-identified or anonymized data. However, when data cannot be publicly shared for ethical reasons, we allow authors to make their data sets available upon request. For information on unacceptable data access restrictions, please see http://journals.plos.org/plosone/s/data-availability#loc-unacceptable-data-access-restrictions.

Before we proceed with your manuscript, please address the following prompts:

a) If there are ethical or legal restrictions on sharing a de-identified data set, please explain them in detail (e.g., data contain potentially identifying or sensitive patient information, data are owned by a third-party organization, etc.) and who has imposed them (e.g., a Research Ethics Committee or Institutional Review Board, etc.). Please also provide contact information for a data access committee, ethics committee, or other institutional body to which data requests may be sent.

We appreciate your stance and are also very pleased to share our data contingent to the slight restrictions mentioned in the paper. They are chosen to comply with EU data protection, ethical requirements and to ensure that data are not used without an analysis plan and other quality measures required by good scientific conduct.

b) If there are no restrictions, please upload the minimal anonymized data set necessary to replicate your study findings to a stable, public repository and provide us with the relevant URLs, DOIs, or accession numbers. Please see http://www.bmj.com/content/340/bmj.c181.long for guidelines on how to de-identify and prepare clinical data for publication. For a list of recommended repositories, please see https://journals.plos.org/plosone/s/recommended-repositories. You also have the option of uploading the data as Supporting Information files, but we would recommend depositing data directly to a data repository if possible.

Please see answer to 5a above.

Please update your Data Availability statement in the submission form accordingly.

6. When completing the data availability statement of the submission form, you indicated that you will make your data available on acceptance. We strongly recommend all authors decide on a data sharing plan before acceptance, as the process can be lengthy and hold up publication timelines. Please note that, though access restrictions are acceptable now, your entire data will need to be made freely accessible if your manuscript is accepted for publication. This policy applies to all data except where public deposition would breach compliance with the protocol approved by your research ethics board. If you are unable to adhere to our open data policy, please kindly revise your statement to explain your reasoning and we will seek the editor's input on an exemption. Please be assured that, once you have provided your new statement, the assessment of your exemption will not hold up the peer review process.

Our data statement is presented in the manuscript and is not contingent upon “acceptance” but upon requests from researchers. Note that the trial is currently ongoing.

7. Please amend the manuscript submission data (via Edit Submission) to include authors J. Hipp, T. Bley, J. Sporn, F. Peters, P. Téoule, and B. Emrullah.

We added the authors accordingly.

8. Please include captions for your Supporting Information files at the end of your manuscript, and update any in-text citations to match accordingly. Please see our Supporting Information guidelines for more information: http://journals.plos.org/plosone/s/supporting-information.

We added the captions accordingly.

9. If the reviewer comments include a recommendation to cite specific previously published works, please review and evaluate these publications to determine whether they are relevant and should be cited. There is no requirement to cite these works unless the editor has indicated otherwise.

Comments to the Author

1. Does the manuscript provide a valid rationale for the proposed study, with clearly identified and justified research questions?

The research question outlined is expected to address a valid academic problem or topic and contribute to the base of knowledge in the field.

Reviewer #1: Partly

Reviewer #2: Yes

Reviewer #3: Yes

2. Is the protocol technically sound and planned in a manner that will lead to a meaningful outcome and allow testing the stated hypotheses?

The manuscript should describe the methods in sufficient detail to prevent undisclosed flexibility in the experimental procedure or analysis pipeline, including sufficient outcome-neutral conditions (e.g. necessary controls, absence of floor or ceiling effects) to test the proposed hypotheses and a statistical power analysis where applicable. As there may be aspects of the methodology and analysis which can only be refined once the work is undertaken, authors should outline potential assumptions and explicitly describe what aspects of the proposed analyses, if any, are exploratory.

Reviewer #1: Yes

Reviewer #2: Yes

Reviewer #3: Yes

3. Is the methodology feasible and described in sufficient detail to allow the work to be replicable?

Descriptions of methods and materials in the protocol should be reported in sufficient detail for another researcher to reproduce all experiments and analyses. The protocol should describe the appropriate controls, sample size calculations, and replication needed to ensure that the data are robust and reproducible.

Reviewer #1: Yes

Reviewer #2: No

Reviewer #3: Yes

4. Have the authors described where all data underlying the findings will be made available when the study is complete?

The PLOS Data policy requires authors to make all data underlying the findings described in their manuscript fully available without restriction, with rare exception, at the time of publication. The data should be provided as part of the manuscript or its supporting information, or deposited to a public repository. For example, in addition to summary statistics, the data points behind means, medians and variance measures should be available. If there are restrictions on publicly sharing data—e.g. participant privacy or use of data from a third party—those must be specified.

Reviewer #1: Yes

Reviewer #2: Yes

Reviewer #3: Yes

5. Is the manuscript presented in an intelligible fashion and written in standard English?

PLOS ONE does not copyedit accepted manuscripts, so the language in submitted articles must be clear, correct, and unambiguous. Any typographical or grammatical errors should be corrected at revision, so please note any specific errors here.

Reviewer #1: Yes

Reviewer #2: Yes

Reviewer #3: Yes

6. Review Comments to the Author

Please use the space provided to explain your answers to the questions above and, if applicable, provide comments about issues authors must address before this protocol can be accepted for publication. You may also include additional comments for the author, including concerns about research or publication ethics.

You may also provide optional suggestions and comments to authors that they might find helpful in planning their study.

(Please upload your review as an attachment if it exceeds 20,000 characters)

Reviewer #1: As the statistical reviewer I will focus on methods and reporting.

Thank you for the careful review.

Major

1) The power calculations do not account for the multicentre nature of the trial. Currently the ICC is expected to be zero. is that a reasonable assumption?

We believe that the case-mix and effect size will be similar between sites, leading to small ICC. Moreover, the primary analysis model takes covariates into account that could account for observed differences between the sites. We have added the following sentence to the manuscript to make this explicit, including a new citation: “Note that the sample size calculation assumes that the intraclass correlation coefficient (ICC) between trial sites is negligible, which has little impact as long as ICC < 0.05 (Islam & Bangidwala 2024). Particularly since relevant covariates are included in the analysis model, this assumption is expected to hold well.”

2) how are missing data implicitly dealt by the mixed effects model? as currently described, it is a complete case analysis, unless there is a Bayesian element to the model and there is data augmentation at play. So some clarity is needed and ideally some information on the use of multiple imputation techniques, if relevant, as I would expect them to be.

We are now more explicit about this point and have written “Missing data are accounted for implicitly by the mixed model in the sense that all patients who provide data at at least one point in time are retained in the model and contribute to the estimates at each point in time. Note that for a mixed model and multiple imputation method using the same analysis model, estimates will be similar asymptotically and the mixed model will be more efficient (Mallinckrodt et al 2020).”

3) if some centres recruit very few patients they may need to be excluded from analyses as a sensitivity, if their inclusion imbalances the estimated random intercepts for centre.

Good idea. Alternatively, or additionally, one could create a fictional site lumping together such patients, but we did not wish to include a fourth sensitivity analysis. We have added the sentence “A third sensitivity analysis will repeat the primary one after excluding patients from sites with very few patients, since their inclusion could affect the estimates of the random term unduly.” and will include this in an amendment of the trial protocol.

Minor

1) the proportional hazards assumption in the Cox model needs to be evaluated.

We note in the paper that the intervention is not expected to affect mortality and therefore would be very surprised if the proportional hazards assumption is violated. We have added the sentence fragment “after ensuring that the proportional hazards assumption is reasonable”

2) the gold standard for anlaysing continuous outcomes in time to even data where mortality is a potential concern, is joint models - discuss as a limitation if you don't conduct, or as a sensitivitiy. Excluding deaths creates immortal time bias and deaths need to be closely monitored and reported to ensure balance across arms.

We added the following sentence to our analysis section “This is a principal stratum analysis for survivors based on the reasonable assumption that the therapy does not affect survival.” If you think of the approach from Rubin for such an analysis, we assume there are no patients who would survive on experimental treatment, but not placebo or vice versa. In the principal stratum, we are not interested in those who die (since by assumption they would die on either treatment) and the analysis as planned is an unbiased estimate of those who survive, since they are expected to survive on either treatment.

Reviewer #2: Hello, this is an interesting and important area of research that has the potential to impact thousands of patients lives.

Please see comments below.

There are several areas where clarification of the study protocol and methods and well as justification for decisions are required. I hope these comments are useful.

Lines 78-80 – please can you check the wording on this? as 5yr mortality rates for gastric cancer are around 24%. Do you mean gastric cancer deaths as a whole?

We revised this passage to more accurately differentiate between perioperative mortality rates and survival rates in gastric cancer treatment.

Lines: 82-84 - ? reference.

The corresponding reference is already provided.

Lines 124 – 129: can this be re-worded to include the specific aim of the trial for clarity

We amended this section as recommended.

Line: 136 – ‘approximately’ rather than ‘about’

We changed it accordingly.

Lines: 156 – 160 – can there be further clarification with the inclusion criteria? Is this all patients – cancer/bariatric surgery/emergency surgery e.g., trauma, GOO etc,

We changed this section as follows: “Having provided written informed consent, the PANEM trial enrolls all adult patients who have undergone total or partial gastrectomy, irrespective of the underlying indication for surgery. Given that gastric cancer represents the predominant indication for gastrectomy, the majority of the study population is expected to comprise patients with oncological diagnoses. However, patients who have undergone gastrectomy for other etiologies are equally eligible for inclusion”.

Table 1 – you have stated ‘key’ inclusion criteria – is this all exclusion criteria or just the main ones? Is just the main ones please list all exclusion criteria

Table 1 now includes all exclusion criteria.

Lines 163 – 167 – please review for clarity. How long post gastrectomy can patients be consented and therefore started on trial? You have stated ‘no later than three days after beginning oral nutrition’ – is this for consenting or starting PERT/placebo? Do you have plans for what happens if oral nutrition is delayed? E.g., patients require PN post surgery – can they still be included?

Thank you for raising this point. Please note, that in a recent protocol amendment, the timeframe for screening and consenting has been extended from three to seven days after the initiation of oral nutrition.

Apart from that both screening and consenting are to be completed within this seven-day window, and PERT/placebo is to be started no later than seven days after the beginning of oral nutrition.

Since an interim parenteral nutrition is not an exclusion criterion those patients are also eligible for study participation, provided that screening and consenting are completed within seven days of commencing oral intake.

Lines 170 – 177: Can the units of lipase (lipase units/kg) be stated for the PERT

We have now included the specific composition and dosage per capsule of the study medication in the manuscript.

Lines 182 – 188: Who is going to be advising patients to increase/decrease dosage? Are they blind to the intervention? Is standardised advice being given regarding the timings/correct methos of taking PERT/placebo? Please state if this is / isn’t the case, with justification.

In our trial, participants are able to self-adjust their dosage based on their individual tolerance and symptoms. Any dose modifications are documented accordingly throughout the study period.

All participants are blinded to the intervention, ensuring that neither the decision to adjust the dosage nor the documentation thereof is influenced by knowledge of group allocation.

Regarding standardized advice on the correct method and timing of intake we added a section to the manuscript :“Participants receive structured instructions on the proper administration of the study medication during their first study visit. This ensures that all participants, regardless of group allocation, receive uniform guidance on how and when to take the medication.”

What is the justification for only increasing PERT at 3 weeks?

The dose escalation window of three weeks was chosen deliberately. The early postoperative period after gastrectomy is characterized by rapid adaptation of gastrointestinal function and dietary intake. Tolerance of oral intake is typically achieved within approximately 5 days after gastrectomy, and dietary patterns begin to stabilize within the first weeks.

Current guidelines recommend an initial dose titration based on clinical response, followed by reassessment at a defined time point — typically at 4–8 weeks after PERT initiation. Our three-week titration window aligns with this approach by allowing individualized dose finding (up to a maximum of 15 capsules of Nortase® per day) during the most dynamic phase of postoperative recovery.

You have stated that the exclusion criteria include ‘indication for PERT’. Please state what happens if someone is experiencing steatorrhea/malabsorption symptoms post-op and once consented to the trial? If they are in the control group are they withdrawn from the trial? As it is not ethical to not provide them with PERT? – noted you have an SAE section – this could be clarified in either section.

Thank you for raising this important point. As this is a double-blind trial, neither the investigators nor the participants are aware of group allocation. Therefore, it is not possible to determine whether a participant experiencing steatorrhoea or malabsorption symptoms is receiving the active intervention or placebo.

It should also be noted that, as described in the manuscript, Nortase® itself — containing fungal-derived enzymes — can cause gastrointestinal symptoms such as abdominal discomfort, which may mimic symptoms of malabsorption. This further underscore the importance of maintaining blinding, as such symptoms cannot be reliably attributed to a lack of enzyme supplementation.

Should a participant develop symptoms that are deemed clinically significant and require emergency intervention, unblinding can be performed at any time in accordance with the study protocol. Following unblinding, appropriate treatment — including initiation of pancreatic enzyme replacement therapy — can be commenced without delay.

Lines 199 – 201 – you’ve stated ‘disease related laboratory measurements’ here, but ‘laboratory measurements related to malnutrition and metabolism’ in the abstract. Please change the abstract to match the body of the article.

We have revised the relevant section accordingly.

Line 210: again, reads as nutrition related blood parameters, please re-word e.g., disease related blood parameters (as Hb, alb, total protein are not nutrition-related measures)

We have revised the relevant section accordingly.

Line 216 – how is glucose measured? Fasting? Random?

We have revised the relevant section accordingly.

Lines 209 – 218 – noted Fig 1 / 2 are provided, but a brief summary of when measures will be taken would improve clarity e.g., baseline, week 3, month 3, month 6, etc..

Fig 1 – states screening is max 10 days before gastrectomy until day ?? (image is unclear) – this does not match text (which states ‘not more than 30 days’), when is the final end point – fig1 states every 6m after 6m – for how long? Please state/clarify.

Thank you for this comment. We believe that Figures 1 and 2 together provide a comprehensive overview of the study timeline and assessment schedule. Figure 1 illustrates the overall study flow including screening, randomization, intervention, and follow-up periods, while Figure 2 details the specific time points at which individual outcome measures are collected (baseline, week 3, month 3, month 6, etc.).

The initial protocol specified a maximum of 10 days before gastrectomy. This was subsequently extended to 30 days in a substantial amendment to allow for greater flexibility in clinical practice. The figure has now been updated to reflect this change and is consistent with the text.

We have added a clarifying statement in the manuscript: “Subsequently, participants will be screened for survival every six months via telephone contact until trial termination.”

Lines: 233 – 234 – How are eating habits screened? Screening tool? Food diary etc.

We clarified this point: “Eating habits were assessed using open-ended questions rather than a standardized questionnaire, allowing for a more individualized exploration of participants' dietary behaviors.”

Lines: 235 – 237 – Please review – states medication will be started at randomisation, but that is incorrect, no? as meds started with oral nutrition?

We revised the relevant section accordingly.

245 -246 – is this the only way that medication compliance is being checked? Is this not asked during the telephone visits/reviews? What is the evidence for the reliability of empty medication bottles being a proxy for medications consumed? Please justify.

Thank you for this comment. We do indeed collect more information and have added it to the text: “Moreover, patients are asked to keep a medication diary, which is reviewed at study visits and consulted upon asking them to assess their own adherence to medication.”

Lines 250 – 251 – please review for clarity – ‘if an adverse effect occurs’? – any examples?

Adverse events are addressed in detail in the dedicated section of the manuscript. We have therefore included a reference to this section for further information.

Lines 293 – 299 – details regarding randomisation. Please can additional information be provided regarding randomisation, in particular in relation to the distribution of the PERT/placebo?

Additional details regarding the distribution of PERT and placebo are provided in the subsequent section of the manuscript.

Reviewer #3: Many thanks for sending the protocol for an Investigator-led, Industry-funded RCT of PERT after gastrectomy. Is it well-written and i located the information i would expect. Overall, we need more data in this space and I support the study. I had a few comments which could improve the protocol if addressed:

1. I support the use of the EORTC-QLQ-C30 for the power calculation / primary outcome given it is well-validated in western populations. In a few places it is described as disease-specific (e.g. abstract, methods), which if i have understood things correctly is not accurate. Secondly, there is an opportunity to obtain more granular data about the benefit of PERT with a gastric-specific assessment, like PGSAS-45. Can a gastrectomy-specific QOL score be added as secondary outcome?

We agree that the EORTC QLQ-C30 is a cancer-specific rather than disease-specific instrument, and we have corrected the wording in the abstract and methods section accordingly.

While we acknowledge that the PGSAS-45 may offer greater sensitivity for postgastrectomy symptoms, it has been predominantly validated in Japanese populations, which limits its applicability and comparability in a Western cohort.

Importantly, our study already incorporates two additional instruments that capture gastrointestinal and gastric cancer-specific aspects of quality of life: the Gastrointestinal Quality of Life Index (GIQLI) and the EORTC QLQ-STO22, both of which are well validated in Western populations and provide data on postgastrectomy symptoms and gastrointestinal function. We added a section on this topic in the manuscript.

At this stage of the trial, it is not feasible to add new scales as secondary outcome measures, as this would require a protocol amendment with implications for regulatory approval, ethics review, and data management.

2. I would suggest expanding the exclusion critieria to patients who have had distal GI surgery, or currently have an active distal GI problem. For example patients who have already lost colon or the ileocaecal valve will already have very different baseline QOL compared to others, and certain subgroups e.g. FAP often have gastric surgery after colonic surgery. The effect of losing the pylorus will also be different in patients with distal pathology/surgery. How to handle distal stomas.

Thank you for raising this point. We will take this into consideration for potential subgroup analyses, allowing us to assess whether prior distal GI surgery or pathology influences treatment outcomes.

3. Participant initiation on the protocol is vague and should be more precisley defined. Is the treatment to be commenced as oral intake is commenced? Is the protocol for initiating oral intake uniform between centres? Is the plan to give the treatment only once food initiated? How are complications like leaks to be handled, and is there a safety concern to the anastomosis, of giving oral proteases in the immediate post-operative period?

The protocol does not prescribe a standardized regimen for initiating oral intake postoperatively. This is intentionally left to the discretion of the treating physicians at each centre, reflecting the variability in clinical practice and individual patient recovery. Study medication is commenced once oral intake has been initiated. Screening and consenting are to be completed within seven days of commencing oral nutrition, as specified in the most recent protocol amendment.

Concerns regarding a potential risk to the anastomosis from oral proteases are understandable but not supported by the available evidence that is already included in the manuscript. A randomised controlled trial of pancreatic enzyme supplementation initiated after gastrectomy for gastric cancer reported no increase in anastomotic complications (Catarci et al., 2018). Furthermore, a recent meta-analysis including 1,620 patients from 24 studies after upper gastrointestinal surgery confirmed a favourable safety profile of PERT, with no reports of anastomotic leak attributable to enzyme supplementation (Hall et al. 2024). These findings are consistent with reviews concluding that timely PERT after gastrointestinal surgery is both safe and beneficial (Chaudhary et al. 2019)

7. PLOS authors have the option to publish the peer review history of their article (what does this mean?). If published, this will include your full peer review and any attached files.

If you choose “no”, your identity will remain anonymous but your review may still be made public.

Do you want your identity to be public for this peer review? For information about this choice, including consent withdrawal, please see our Privacy Policy.

Reviewer #1: No

Reviewer #2: No

Reviewer #3: No

Attachment Submitted filename: Response_to_reviewers_2026-06-05_Kevin Thomas.docx

# Peer review: Decision Letter 1

**DOI:** 10.1371/journal.pone.0358177.r003

**Authors:** Banreet Singh Dhindsa

27 Aug 2026

<p>Protocol of the PANEM trial: A randomized, phase III, parallel-arm, placebo-controlled, double-blind, multi-center superiority trial comparing PANcreatic EnzyMes versus placebo in adults after gastrectomy

PONE-D-25-68836R1

Dear Dr. Thomas,

We’re pleased to inform you that your manuscript has been judged scientifically suitable for publication and will be formally accepted for publication once it meets all outstanding technical requirements.

Within one week, you’ll receive an e-mail detailing the required amendments. When these have been addressed, you’ll receive a formal acceptance letter and your manuscript will be scheduled for publication.

An invoice will be generated when your article is formally accepted. Please note, if your institution has a publishing partnership with PLOS and your article meets the relevant criteria, all or part of your publication costs will be covered. Please make sure your user information is up-to-date by logging into Editorial Manager at Editorial Manager® and clicking the ‘Update My Information' link at the top of the page. For questions related to billing, please contact billing support.

If your institution or institutions have a press office, please notify them about your upcoming paper to help maximize its impact. If they’ll be preparing press materials, please inform our press team as soon as possible -- no later than 48 hours after receiving the formal acceptance. Your manuscript will remain under strict press embargo until 2 pm Eastern Time on the date of publication. For more information, please contact onepress@plos.org.

Kind regards,

Banreet Singh Dhindsa

Academic Editor

PLOS One

Additional Editor Comments (optional):

Reviewers' comments:

Reviewer's Responses to Questions

**Comments to the Author**

1. Does the manuscript provide a valid rationale for the proposed study, with clearly identified and justified research questions?

Reviewer #1: Yes

Reviewer #3: Yes

**********

2. Is the protocol technically sound and planned in a manner that will lead to a meaningful outcome and allow testing the stated hypotheses?

Reviewer #1: Yes

Reviewer #3: Yes

**********

3. Is the methodology feasible and described in sufficient detail to allow the work to be replicable?

Reviewer #1: Yes

Reviewer #3: Yes

**********

4. Have the authors described where all data underlying the findings will be made available when the study is complete?

The PLOS Data policy

Reviewer #1: Yes

Reviewer #3: Yes

**********

5. Is the manuscript presented in an intelligible fashion and written in standard English?

Reviewer #1: Yes

Reviewer #3: Yes

**********

Please use the space provided to explain your answers to the questions above and, if applicable, provide comments about issues authors must address before this protocol can be accepted for publication. You may also include additional comments for the author, including concerns about research or publication ethics.

You may also provide optional suggestions and comments to authors that they might find helpful in planning their study.

Reviewer #1: I an satisfied with the author's responses and the resulting changes to the paper. Nothing else to add.

Reviewer #3: Thank you for your work

**********

what does this mean?). If published, this will include your full peer review and any attached files.

If you choose “no”, your identity will remain anonymous but your review may still be made public.

**Do you want your identity to be public for this peer review?**  For information about this choice, including consent withdrawal, please see our Privacy Policy

Reviewer #1: No

Reviewer #3: No

**********

# Editor report: Acceptance letter

**DOI:** 10.1371/journal.pone.0358177.r004

**Authors:** Banreet Singh Dhindsa

PONE-D-25-68836R1

PLOS One

Dear Dr. Thomas,

I'm pleased to inform you that your manuscript has been deemed suitable for publication in PLOS One. Congratulations! Your manuscript is now being handed over to our production team.

At this stage, our production department will prepare your paper for publication. This includes ensuring the following:

* All references, tables, and figures are properly cited

* All relevant supporting information is included in the manuscript submission,

* There are no issues that prevent the paper from being properly typeset

You will receive further instructions from the production team, including instructions on how to review your proof when it is ready. Please keep in mind that we are working through a large volume of accepted articles, so please give us a few days to review your paper and let you know the next and final steps.

Lastly, if your institution or institutions have a press office, please let them know about your upcoming paper now to help maximize its impact. If they'll be preparing press materials, please inform our press team within the next 48 hours. Your manuscript will remain under strict press embargo until 2 pm Eastern Time on the date of publication. For more information, please contact onepress@plos.org.

You will receive an invoice from PLOS for your publication fee after your manuscript has reached the completed accept phase. If you receive an email requesting payment before acceptance or for any other service, this may be a phishing scheme. Learn how to identify phishing emails and protect your accounts at https://explore.plos.org/phishing.

If we can help with anything else, please email us at customercare@plos.org.

Thank you for submitting your work to PLOS One and supporting open access.

Kind regards,

PLOS One Editorial Office Staff

on behalf of

Dr. Banreet Singh Dhindsa

Academic Editor

PLOS One
